# Manipulating the functionality and structures of π-conjugated polymers utilizing intramolecular noncovalent interactions towards efficient organic photovoltaics†

**DOI:** 10.1039/d4sc00899e

**Published:** 2024-03-25

**Authors:** Satoshi Kamimura, Masahiko Saito, Yoshikazu Teshima, Kodai Yamanaka, Hiroyuki Ichikawa, Ai Sugie, Hiroyuki Yoshida, Jihun Jeon, Hyung Do Kim, Hideo Ohkita, Tsubasa Mikie, Itaru Osaka

**Affiliations:** a Department of Applied Chemistry, Graduate School of Engineering, Hiroshima University Higashi-Hiroshima Hiroshima 739-8527 Japan iosaka@hiroshima-u.ac.jp; b Applied Chemistry Program, Graduate School of Advanced Science and Engineering, Hiroshima University Higashi-Hiroshima Hiroshima 739-8527 Japan; c Department of Materials Science, Graduate School of Engineering, Chiba University 1-33 Yayoi-cho, Inage-ku Chiba 263-8522 Japan; d Molecular Chirality Research Center, Chiba University 1-33 Yayoi-cho, Inage-ku Chiba 263-8522 Japan; e Department of Polymer Chemistry, Graduate School of Engineering, Kyoto University Katsura Nishikyo-ku Kyoto 615-8510 Japan

## Abstract

Careful control of electronic properties, structural order, and solubility of π-conjugated polymers is central to the improvement of organic photovoltaic (OPV) performance. In this work, we designed and synthesized a series of naphthobisthiadiazole–quaterthiophene copolymers by systematically replacing the alkyl groups with ester groups and changing the position of the fluorine groups in the quaterthiophene moiety. These alterations lowered the HOMO and LUMO energy levels and systematically varied the combination of intramolecular noncovalent interactions such as O⋯S and F⋯S interactions in the backbone. More importantly, although the introduction of such noncovalent interactions often lowers the solubility owing to the interlocking of backbone linkages, we found that careful design of the noncovalent interactions afforded polymers with relatively high solubility and high crystallinity at the same time. As a result, the power conversion efficiency of OPV cells that used fullerene (PC_61_BM) and nonfullerene (Y12) as the acceptor was improved. Our work offers important information for the development of high-performance π-conjugated polymers for OPVs.

## Abstract 2

We synthesized a series of naphthobisthiadiazole-polymers by systematically introducing the alkyl, ester, and fluorine groups in the quaterthiophene co-unit and studied the effect of noncovalent interactions on physical and photovoltaic properties.

## Graphical abstract

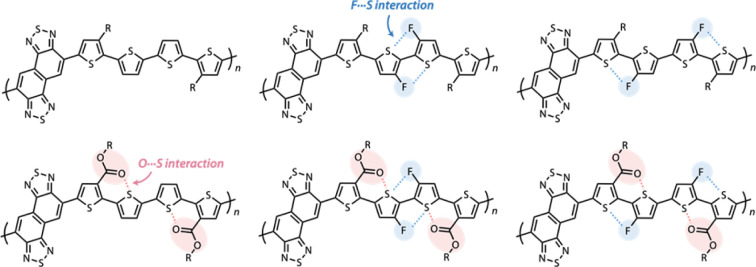

## Introduction

π-Conjugated polymers have been attracting substantial attention owing to their excellent functionality in electronic devices such as organic field-effect transistors (OFETs) and organic photovoltaics (OPVs).^1,2^ In particular, a large number of π-conjugated polymers and OPVs have been reported, which resulted in the power conversion efficiency (PCE) surpassing 19% in single-junction cells when combined with nonfullerene acceptors (NFAs).^3–5^ Because π-conjugated polymers and their blends are solution-processed to form thin films as the active layer of devices, they are required to have good solubility in organic solvents. In the meantime, as backbone coplanarity, π–π interaction and/or crystallinity, and π-conjugated polymer orientation have an impact on charge transport,^6–10^ charge separation,^11–13^ and/or charge recombination,^11,12,14^ these structural orders must be carefully controlled. Furthermore, the energy levels of the highest occupied molecular orbital (HOMO) and the lowest unoccupied molecular orbital (LUMO) are equally crucial as they dictate charge carrier polarity (p-type, n-type, or ambipolar),^15,16^ charge injection,^15^ charge separation,^15–19^ and/or photovoltage.^16,18–21^ These polymer structures and properties are strongly dependent on the chemical structures of the backbone, the side chain, and their combination. However, it is very difficult to balance all these factors; for example, solubility and π–π interaction/crystallinity are often in a trade-off relationship.

In the design of π-conjugated polymers, donor–acceptor type backbones with fused heteroaromatic rings are often employed to enhance backbone coplanarity and π–π interactions, narrow the optical bandgap, and/or tune the energy levels.^15,22–27^ In such donor–acceptor π-conjugated polymers, long, branched alkyl groups are typically introduced as the side chain to ensure solubility. Alkoxy and ester groups with a branched alkyl moiety are also introduced often as the solubilizing group. In addition, because of electronic effects, these heteroatom-containing groups and halogens such as fluorine are often introduced as substituents as they play a vital role in tuning the energetics. Furthermore, these heteroatom-containing groups and halogens induce intramolecular noncovalent interactions with the sulfur of thiophene or thiophene-related rings and suppress the torsion of the linkage and thus the polymer backbone, leading to strong π–π interactions.^28–33^ Therefore, it is important to carefully combine the donor–acceptor backbone with these functional groups in addition to or instead of the alkyl groups to manipulate the solubility, energy levels, and structural orders of π-conjugated polymers, thereby maximizing device performance.

**Fig. 1:**
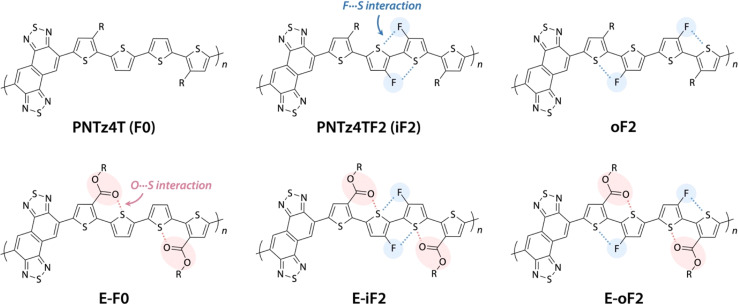
Chemical structures of naphthobisthiadiazole-based polymers studied in this work.

We have been focusing on a series of π-conjugated polymers based on naphthobisthiadiazole (NTz), such as PNTz4T (Fig. 1), because they possess high crystallinity and exhibit relatively high OFET and OPV performances,^34–36^ while several other groups have also reported on NTz-based π-conjugated polymers.^37–39^ Recently, we reported that the introduction of fluorine groups onto the PNTz4T backbone lowered the HOMO and/or LUMO energy levels depending on the substitution position and enhanced crystallinity, which resulted in the high PCE of the OPV device.^40,41^ However, as mentioned above, the fluorination significantly decreased the solubility owing to the intramolecular noncovalent interaction between the fluorine atom substituted on the thiophene ring and the sulfur atom of the neighboring thiophene ring (F⋯S interaction), giving rise to the loss of processability. These results propelled us to seek ways to manage the solubility, energy levels, and structural orders of π-conjugated polymers using the NTz polymer system as the platform.

**Fig. 2:**
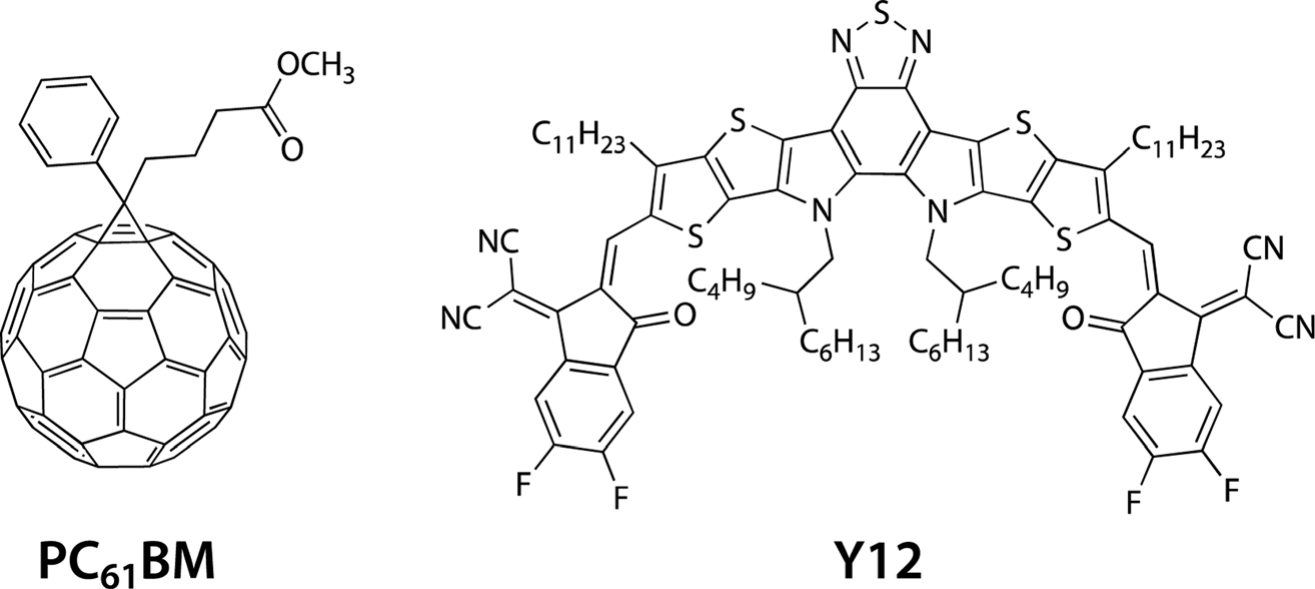
Chemical structures of PC_61_BM and Y12 used as acceptor materials for OPV devices.

Here, we study a series of PNTz4T derivatives consisting of NTz and the quaterthiophene moiety, in which fluorine groups and/or ester groups are introduced onto the quaterthiophene moiety, as displayed in Fig. 1. PNTz4T, as we have reported previously, has long and branched alkyl groups as the side chain on the thiophene rings that neighbor the NTz unit.^34–36^ In order to simplify and better distinguish the polymers, PNTz4T is hereinafter called F0. The fluorinated counterparts have fluorine atoms added to the central thiophene rings of the quaterthiophene moiety at the “inner” and “outer” β-positions and are thus named iF2 (formerly named PNTz4TF2)^40^ and oF2, respectively. oF2 is newly synthesized in this work. E-F0, in which the alkyl groups of F0 are replaced by ester groups, is also newly synthesized. Similar to the alkylated polymers, fluorine groups are also introduced onto the thiophene rings in E-F0, and the resulting polymers are called E-iF2 and E-oF2. As both fluorine and ester groups have an electron-withdrawing nature, the introduction of these groups effectively lowers the energy levels. Furthermore, the fluorine and the carbonyl oxygen of the ester group cause noncovalent interactions with sulfur of the neighboring thiophene ring (F⋯S and O⋯S interactions) (Fig. 1), which can interlock the linkage between the neighboring heteroaromatic rings and in turn, coplanarize the backbone. We investigate and discuss the correlation between the molecular structure and the solubility, electronic properties, and structural order in association with simple quantum chemical calculations using models. We also fabricate OPV devices using PC_61_BM as a fullerene acceptor and Y12 (ref. 42) as an NFA (Fig. 2) and discuss how these functionalities and structures influence device photovoltaic performance.

## Results and discussion

### Synthesis

**Scheme 1:**
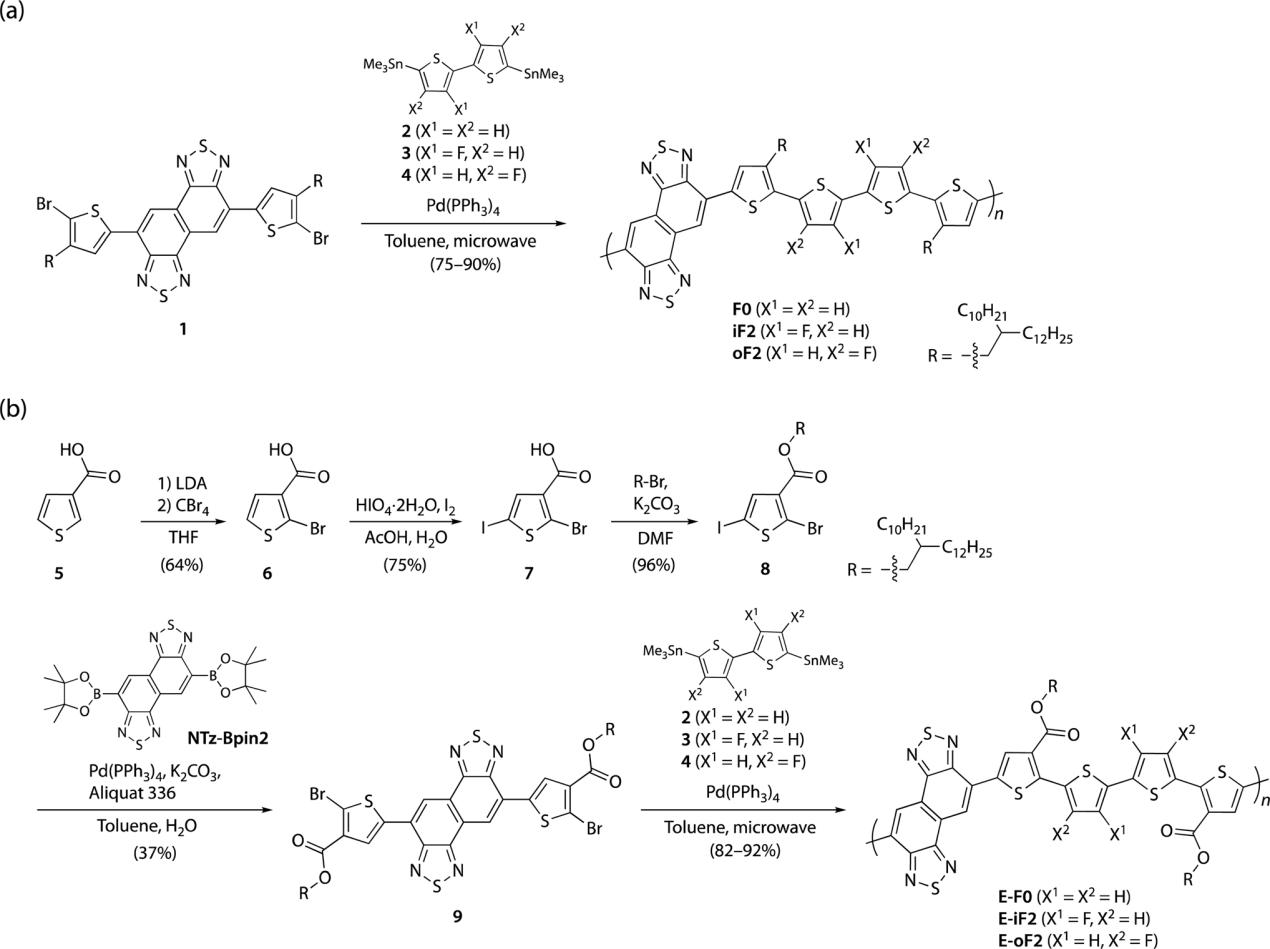
(a) Synthesis of polymers with alkyl side chains F0, iF2, and oF2. (b) Synthesis of polymers with ester side chains E-F0, E-iF2, and E-oF2.

Scheme 1a and b illustrate the synthesis of polymers with alkyl and ester side chains, respectively. F0 and iF2 were synthesized following the reported procedures,^33,40^ in which NTz monomer 1 was copolymerized with 5,5′-bis(trimethylstannyl)-2,2′-bithiophene (2) and 5,5′-bis(trimethylstannyl)-3,3′-difluoro-2,2′-bithiophene (3), respectively, *via* the Migita–Kosugi–Stille cross-coupling reaction. oF2 was newly synthesized similarly by using 1 and 5,5′-bis(trimethylstannyl)-4,4′-difluoro-2,2′-bithiophene (4).^43^ For the polymers with ester side chains, the ester-substituted NTz monomer was first synthesized as follows. 3-Thiophenecarboxylic acid (5) was brominated at the 2-position through lithiation by lithium diisopropyl amide (LDA) and the following treatment with tetrabromomethane to yield 2-bromo-3-thiophenecarboxylic acid (6). 6 was then iodinated using periodic acid and iodine to give 2-bromo-5-iodo-3-thiophenecarboxylic acid (7), and 7 was esterified to provide 8 using bromo-2-decyltetradecane in the presence of potassium carbonate. The Suzuki–Miyaura cross-coupling reaction of 8 and diborylated NTz (NTz-Bpin2) afforded ester-substituted NTz monomer 9. Finally, 9 was copolymerized with 2, 3, and 4, yielding E-F0, E-iF2, and E-oF2, respectively.

The molecular weights of the polymers were evaluated by high-temperature gel permeation chromatography (GPC) (Fig. S1†). All the polymers had sufficiently high molecular weights; the number-average molecular weight (*M*_n_) was more than 30 000 and the dispersity (Đ) ranged from around 2 to 4 (Table 1). The thermal properties of the polymers were investigated by differential scanning calorimetry (DSC) (Fig. S2†). Whereas F0 and oF2 showed a peak indicative of melting at around 300 °C, other polymers did not show any peaks. The results suggest that the polymers are thermally stable.

**Table 1:** Molecular weights and dispersity values of the polymers^a^

Polymer	*M*_n_	*M*_w_	Đ (*M*_w_/*M*_n_)
F0	37 300	81 800	2.20
iF2	53 500	237 000	4.43
oF2	33 800	65 900	1.95
E-F0	124 000	241 000	1.94
E-iF2	44 500	100 000	2.25
E-oF2	43 800	96 500	2.20

a Molecular weights were determined by high-temperature GPC at 140 °C using *o*-dichlorobenzene (DCB) as the eluent and calibrated using a polystyrene standard.

### Polymer electronic properties

**Fig. 3:**
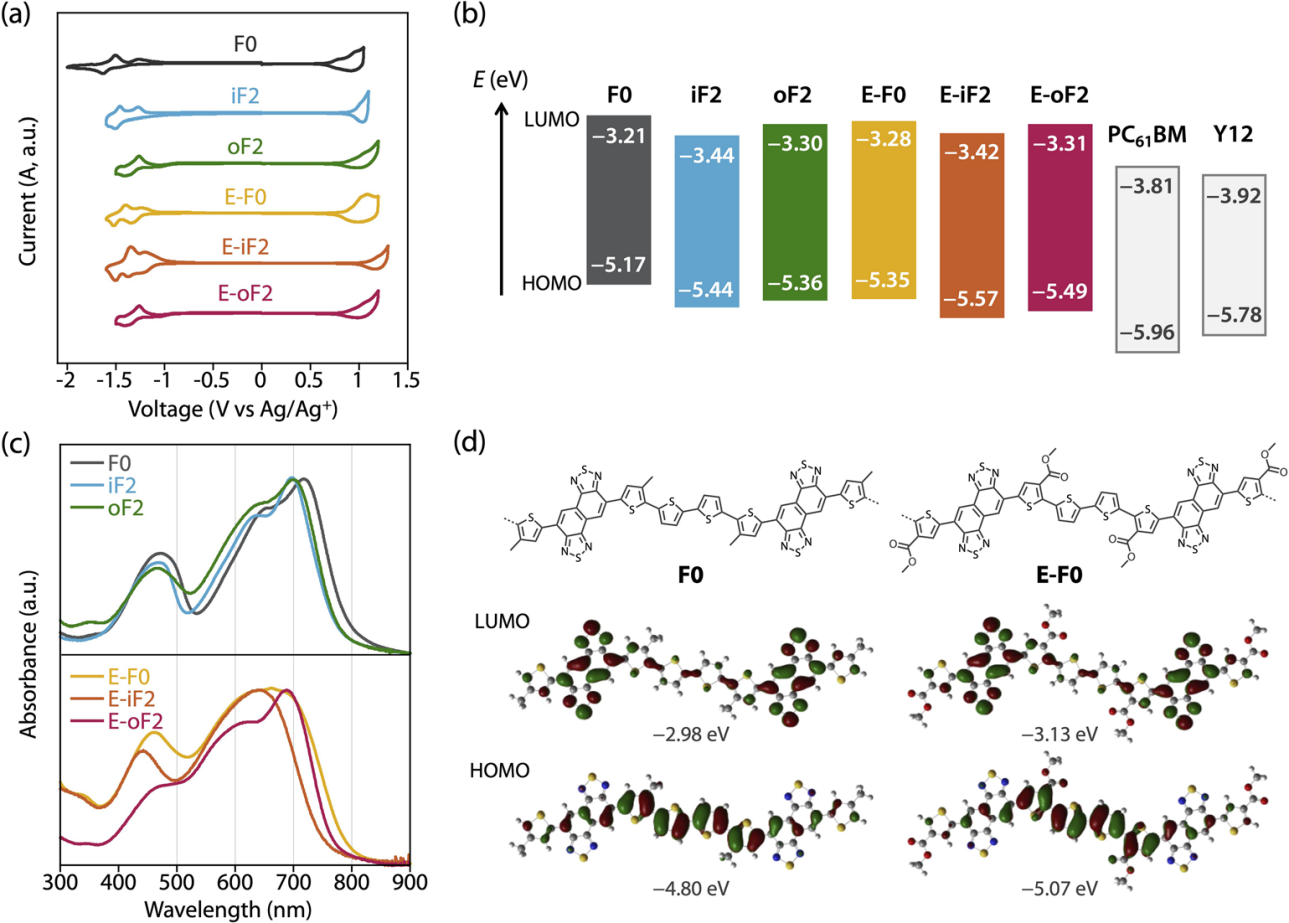
(a) Cyclic voltammograms of the polymer thin films. (b) Energy diagrams of the polymers and acceptors. (c) UV-vis absorption spectra of the polymers in the thin film. (d) Geometry of HOMO and LUMO for the models of F0 and E-F0.

The HOMO and LUMO energy levels (*E*_HOMO_ and *E*_LUMO_) of the polymers were investigated by cyclic voltammetry (CV) (Fig. 3a) and are summarized in Table 2 as well as Fig. 3b. *E*_HOMO_ and *E*_LUMO_ were also estimated by photoelectron yield spectroscopy and low-energy inverse photoelectron spectroscopy,^44,45^ respectively (Fig. S3 and Table S1†), and were mostly consistent with those estimated by CV. The exciton binding energies determined from *E*_HOMO_, *E*_LUMO_, and the optical bandgap (*E*^opt^_g_) estimated from the film absorption spectrum as shown later are in the range of 0.4–0.5 eV (Table S1†). These values are a quarter of the transport gaps (*E*_HOMO_ – *E*_LUMO_), which is consistent with our previous observation.^46^ The *E*_LUMO_ and *E*_HOMO_ of F0 were −3.21 eV and −5.17 eV, respectively, which were lowered to −3.44 eV and −5.44 eV when fluorine atoms were introduced onto the inner β-positions of the bithiophene moiety (iF2). The down shift of *E*_HOMO_ was larger than that of *E*_LUMO_. This can be understood from the LUMO and HOMO geometries of F0, where the density of HOMO was higher than that of LUMO in the quaterthiophene moiety, most likely due to the electron-rich nature (Fig. 3d, see Fig. S4† for the LUMO and HOMO geometries of all the polymer models). For oF2 having fluorine atoms at the outer *β*-positions, *E*_LUMO_ and *E*_HOMO_ were −3.30 eV and −5.36 eV, respectively, which were lower than those of F0 but higher than those of iF2.

**Table 2:** Electronic properties of the polymers

Polymer	*E*_LUMO_ a (eV)	*E*_HOMO_ b (eV)	Δ*E*_L_ (eV)	Δ*E*_H_ (eV)	*λ*_max_ c (nm)	*λ*_onset_ d (nm)	*E* ^opt^_g_ e (eV)
F0	−3.21	−5.17	0.60	0.61	718	800	1.55
iF2	−3.44	−5.44	0.37	0.34	700	770	1.61
oF2	−3.30	−5.36	0.51	0.42	699	772	1.61
E-F0	−3.28	−5.35	0.53	0.43	662	795	1.56
E-iF2	−3.42	−5.57	0.39	0.21	622	743	1.67
E-oF2	−3.31	−5.49	0.50	0.29	689	768	1.61

a LUMO energy levels determined by cyclic voltammetry (CV).

b HOMO energy levels determined by CV.

c Absorption maximum.

d Absorption onset.

e Optical band gap determined from the absorption onset.

E-F0 having ester side chains exhibited the *E*_LUMO_ and *E*_HOMO_ of −3.28 eV and −5.35 eV, which were lower than and similar to F0, respectively. This can be explained by the fact that the electron-withdrawing ester groups were introduced onto the thiophene rings where the density of HOMO was higher than that of LUMO (Fig. 3d). E-iF2 and E-oF2 provided *E*_LUMO_s of −3.42 eV and −3.31 eV and *E*_HOMO_s of −5.57 eV and −5.49 eV, respectively, which were lower than those of E-F0. This trend was consistent with that observed in alkylated polymers.

As such, the energy levels of the polymers were lowered by the introduction of fluorine atoms and the replacement of the alkyl groups with ester groups, which corresponded to the HOMO and LUMO density of the substituted positions. In addition, we also determined the energy levels of the acceptor materials used in this study, PC_61_BM and Y12, by CV (Fig. S5†). The *E*_LUMO_ and *E*_HOMO_ of PC_61_BM were −3.81 eV and −5.96 eV, respectively, and therefore the LUMO offset energy (Δ*E*_L_) values between the polymer and PC_61_BM for all the blends were sufficiently large (0.4–0.6 eV) to allow electron transfer from the polymer to PC_61_BM. The *E*_LUMO_ and *E*_HOMO_ of Y12 were found to be −3.92 eV and −5.78 eV, respectively. The HOMO offset energy (Δ*E*_H_) between the polymer and Y12 was 0.34–0.6 eV for the alkylated polymers and E-F0, which should be sufficiently large to allow hole transfer from Y12 to the polymers. However, E-F0 and E-oF2 had slightly small Δ*E*_H_, 0.21 eV and 0.29 eV, respectively, which could be detrimental to the hole transfer. These offset energies were consistent with the photoluminescence (PL) quenching efficiencies as will be described later.

The UV-vis absorption spectra of the polymers in the thin films were measured (Fig. 3c). F0 had an absorption maximum (*λ*_max_) at 718 nm and a shoulder at around 650 nm, which were assigned to the 0–0 and 0–1 transition bands, respectively. In addition, the absorption onset (*λ*_onset_) was 800 nm, and this value corresponded to an energy (optical bandgap: *E*^opt^_g_) of 1.55 eV. The absorption spectrum of iF2 slightly sharpened compared with that of F0, most likely owing to the enhanced backbone coplanarity originating from the F⋯S interactions. The absorption band of iF2 was also slightly blue-shifted, resulting in a *λ*_max_ of 700 nm and a *λ*_onset_ of 770 nm, and thus an *E*^opt^_g_ of 1.61 eV. The absorption band of oF2 slightly broadened toward the short-wavelength region compared with that of iF2, although *λ*_max_ (699 nm) and *E*^opt^_g_ (1.61 eV) were the same. This suggests that the coplanarity and/or ordering of a single polymer chain for oF2 is slightly lower than that for iF2. This is likely due to the difference in the fluorine substitution position and thus the difference in the location of F⋯S interactions, which will be discussed later.

E-F0 gave a broad featureless spectrum in contrast to the alkyl counterpart F0, suggesting that the polymer backbone was less coplanar and/or less ordered, even though there were O⋯S interactions between the carbonyl oxygen and sulfur. Although the *λ*_max_ of E-F0 (662 nm) was blue-shifted from that of F0 (718 nm), the *E*^opt^_g_ (1.56 eV) of E-F0 was similar to that of F0 (1.55 eV) owing to the broad absorption spectrum. E-iF2 also showed a featureless absorption spectrum, but it was narrower than that of E-F0. This is probably due to the enhanced backbone coplanarity by the F⋯S interactions. Interestingly, E-oF2 provided a relatively sharp spectrum with a clear 0–0 transition band and a 0–1 transition band as the shoulder. This implies that the backbone of E-oF2 is more coplanar and ordered than that of E-iF2 and sharply contrasts with the case between iF2 and oF2, although the origin is unclear so far.

### Effects of the motif and location of noncovalent interactions

**Fig. 4:**
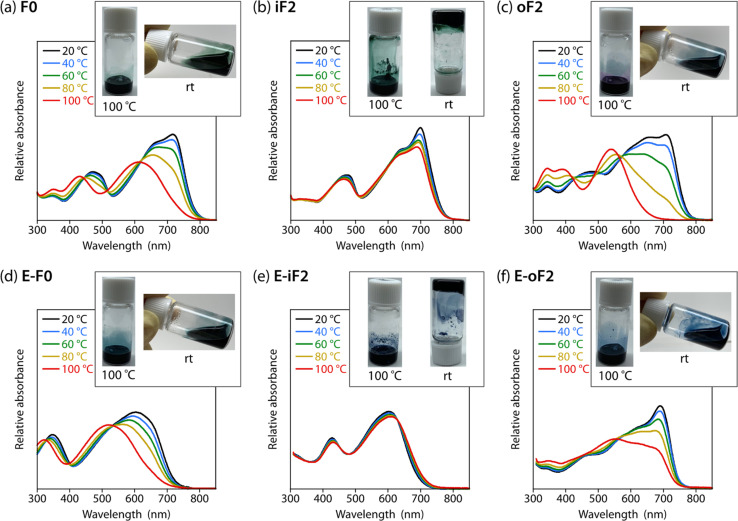
Temperature dependence of the absorption spectra of the polymer solutions in DCB. Insets show photographs of the polymer solutions in DCB heated up to 100 °C and cooled down to room temperature after complete dissolution. (a) F0, (b) iF2, (c) oF2, (d) E-F0, (e) E-iF2, and (f) E-oF2.

To study the effects of the motif and the location of noncovalent F⋯S and O⋯S interactions on the backbone rigidity, aggregation properties, and the solubility, we recorded the temperature-dependent absorption spectra of the polymers in *o*-dichlorobenzene (DCB) solution (Fig. 4). The absorption band of F0 was gradually blue-shifted as the temperature was increased from 20 to 100 °C, and finally became featureless (Fig. 4a), suggesting that the heating induced torsion and disaggregation of the backbone. In iF2, the change in the absorption spectrum was much smaller than that in F0. This suggests that the backbone is more rigid and has stronger aggregation properties owing to the two F⋯S interactions in the bithiophene moiety (Fig. 4b), which interlock the thiophene–thiophene linkage and suppress the bond rotation. In contrast to iF2, oF2 showed a drastic blueshift of the absorption spectrum as the temperature was increased despite having F⋯S interactions (Fig. 4c); the blueshift was even more significant than that in F0. This indicates that the interlocking strength is much weaker in oF2 than in iF2. In E-F0, although there are two O⋯S noncovalent interactions between the carbonyl oxygen of the ester group and the sulfur of the neighboring thiophene ring, the absorption spectrum showed a marked blueshift as the temperature was increased (Fig. 4d), as was observed in the case of F0. E-iF2 did not show a clear shift of the absorption spectrum (Fig. 4e), whereas E-oF2 showed a significant blueshift as the temperature was increased (Fig. 4f). Thus, the trends observed in E-iF2 and E-oF2 were similar to those observed in iF2 and oF2. It should be noted that in E-oF2, the absorption spectrum did not show a complete blueshift at 100 °C as in the case of oF2, probably because of the enhanced backbone rigidity originating in the addition of the O⋯S interactions to the F⋯S interactions.

We then tested the solubility by dissolving the polymer samples in DCB at the concentration of 5 g L^−1^. The mixture of the polymer and DCB was heated until the polymer completely dissolved and then cooled to room temperature. Photographs of the solution heated up to 100 °C and cooled down to room temperature after complete dissolution are shown in Fig. 4 as the inset of each temperature-dependent absorption spectrum. F0 dissolved completely at 100 °C and remained dissolved even after the cooling to room temperature (Fig. 4a). In contrast, iF2 dissolved at approximately 140 °C (it did not dissolve completely at 100 °C) and solidified to form a gel at room temperature (Fig. 4b). oF2 dissolved at 100 °C and remained dissolved at room temperature, similar to F0. For the ester-substituted polymers, whereas E-F0 and E-oF2 dissolved at 100 °C and remained dissolved at room temperature (Fig. 4d and f) as observed in F0 and oF2, E-iF2 dissolved at around 140 °C and formed a gel after the cooling to room temperature (Fig. 4e), as observed in iF2. Nevertheless, the ester-substituted polymers had slightly lower solubility than the corresponding alkylated polymers; E-F0, E-iF2, and E-oF2 were slightly less soluble in DCB than F0, iF2, and oF2, respectively. The results of the solubility test are in good agreement with the temperature-dependent absorption spectra. The molecular weights of the polymers are sufficiently high as shown above and there would be no molecular-weight effect on the solubility difference. Thus, careful choice of the substitution position of the fluorine atom and/or the substitution of the ester group does not necessarily decrease the solubility of the π-conjugated polymers significantly even though they induce interlocking in the backbone.

### Understanding polymer rigidity and solubility

**Fig. 5:**
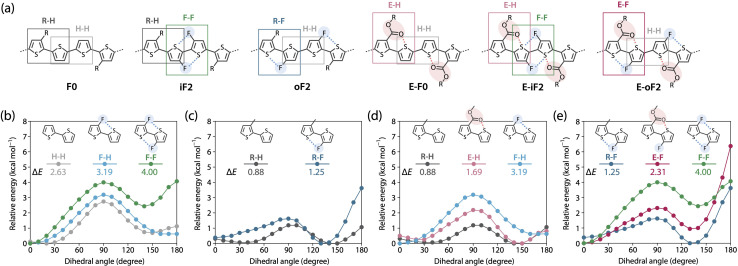
(a) The six different quaterthiophene moieties of the polymers. (b–d) Energy variations of thiophene–thiophene linkages with various substituent combinations as a function of dihedral angle. Comparison of (b) thiophene–thiophene (H–H), fluorothiophene–thiophene (F–H), and fluorothiophene–fluorothiophene (F–F); (c) methylthiophene–thiophene (R–H) and methylthiophene–fluorothiophene (R–F); (d) R–H, esterthiophene–thiophene (E–H), and F–H; and (e) R–F, esterthiophene–fluorothiophene (E–F), and F–F. The corresponding linkages are highlighted with squares in the quaterthiophene moieties shown in (a).

To further understand differences in the solubility and the backbone rigidity and order of this polymer series, we investigated the relative energy of the thiophene–thiophene linkage for various bithiophene derivatives with different substituent combinations using methyl, fluorine, and methyl ester groups as a function of the dihedral angle (*θ*), by DFT calculation (Fig. 5). Fig. 5a displays the six different quaterthiophene moieties corresponding to the present polymers, which show a combination of corresponding thiophene–thiophene model linkages: thiophene–thiophene (H–H), fluorothiophene–fluorothiophene (F–F), methylthiophene–thiophene (R–H), methylthiophene–fluorothiophene (R–F), esterthiophene–thiophene (E–H), and esterthiophene–fluorothiophene (E–F) linkages. Here, the coplanar form with the *anti*-configuration was defined as *θ* = 0°, and the difference between the energies at *θ* = 0° and *θ* = 90° was defined as the rotational barrier (Δ*E*) of the linkage. The Δ*E* values are shown in the plots.

First, we show the plots for H–H, fluorothiophene–thiophene (F–H), and F–F linkages (Fig. 5b), and discuss the effect of fluorination at the inner *β*-positions of the bithiophene moiety in the polymers. With the F⋯S interaction, Δ*E* for F–H (3.19 kcal mol^−1^) was larger than that for H–H (2.63 kcal mol^−1^), and with the two F⋯S interactions, Δ*E* for F–F (4.00 kcal mol^−1^) was the largest. Notably, the difference in Δ*E* between F–F and F–H was greater than that between F–H and H–H. In addition, for F–F, the coplanar form with the *syn*-configuration (*θ* = 180°) had a large energy that was comparable to that of the twisted form (*θ* = 90°), rendering it significantly unstable; this indicates that F–F prefers the coplanar *anti*-configuration (*θ* = 0°) more than the coplanar *syn*-configuration. This sharply contrasted with the case of H–H and F–H, in which the coplanar *syn*-configuration was comparably stable with the *anti*-configuration. This is probably because of the steric and/or possibly electrostatic repulsions between the fluorine atoms in the *syn*-configuration. Therefore, difluorination in the bithiophene moiety had an extremely high impact on linkage interlocking, resulting in high aggregation properties. This agrees well with the fact that iF2 (R–H/F–F) and E-iF2 (E–H/F–F) had much lower solubility than F0 (R–H/H–H) and E-F0 (E–H/H–H), respectively.

In Fig. 5c, we have plotted the energy variation of R–H and R–F linkages. On the basis of this comparison, we discuss the effect of fluorination at the outer *β*-positions of the bithiophene moiety in the polymers. Δ*E* for R–F (1.25 kcal mol^−1^) was slightly larger than that for R–H (0.88 kcal mol^−1^), and this tendency was similar to that observed for H–H and F–H. Moreover, Δ*E* for R–F and R–H was smaller than that for H–H and H–F, respectively, which could be ascribed to the steric repulsion from the methyl group. Notably, Δ*E* for R–F was smaller than that for H–H without noncovalent interactions, meaning that the linkage in R–F can be relatively easily rotated even though it has an F⋯S interaction. However, because the *syn*-configuration was significantly more unstable in R–F than in R–H, most likely owing to the steric repulsion between the methyl and fluorine groups, R–F would prefer to form the *anti*-configuration. Thus, the fluorine atom acts as a conformational-directing group in this case: the fluorine substitution can induce the *anti*-configuration except for F–H linkages. The results in Fig. 5b and c strongly indicate that the rigidity and solubility of oF2 (R–F/H–H) were significantly lower and higher than those of iF2 (R–H/F–F), respectively, and were similar to those of F0 (R–H/H–H), and that the backbone order of oF2 was similar to that of iF2 and F0.

Fig. 5d shows the energy variation of R–H, E–H, and F–H linkages. We examined the effect of replacing the alkyl group with an ester group and the difference in the strength between O⋯S and F⋯S interactions. Δ*E* for E–H (1.69 kcal mol^−1^) was larger than that for R–H (0.88 kcal mol^−1^) likely because of the O⋯S interaction. Seemingly, however, the difference in Δ*E* was somewhat greater than expected as E-F0 (E–H/H–H) showed only slightly lower solubility than F0 (R–H/H–H). The alkoxy moiety in the ester group could freely rotate, and this might have increased the solubility. The lower Δ*E* for E–H than for F–H suggests that the O⋯S interaction has a weaker interlocking effect than the F⋯S interaction, likely because the pseudo-five-membered ring is formed when the F⋯S interaction exists in F–H whereas the pseudo-six-membered ring is formed when the O⋯S interaction exists in E–H. Fig. 5e summarizes the energy variation of R–F, E–F, and F–F linkages. Similar to the trend between R–H and E–H, Δ*E* for E–F (2.31 kcal mol^−1^) was larger than that for R–F (1.25 kcal mol^−1^) owing to the additional O⋯S interaction. It is noted that Δ*E* for E–F was significantly smaller than that for F–F even though both have two noncovalent interactions. This again shows the difference in the interlocking effect between the O⋯S and F⋯S interactions. On the other hand, a striking feature in E–F was that the instability of the *syn*-configuration was greater than the other linkages, and thus the *anti*-configuration should be highly preferred. Therefore, similar to the case of R–F, the fluorine atom acts as a conformational-directing group in E–F linkages.

Overall, these calculations furnish useful information on linkage characteristics and reasonably explain the solubility and the backbone rigidity and order of these polymers. For example, the polymer having fluorines at the bithiophene “outer” positions such as oF2 (R–F/H–H) showed higher solubility and lower rigidity, but similar or higher backbone order than that at the bithiophene “inner” positions such as iF2 (R–H/F–F), and ester-substituted polymers exhibited similar solubility to the corresponding alkyl-substituted polymers.

### Polymer order in neat film

**Fig. 6:**
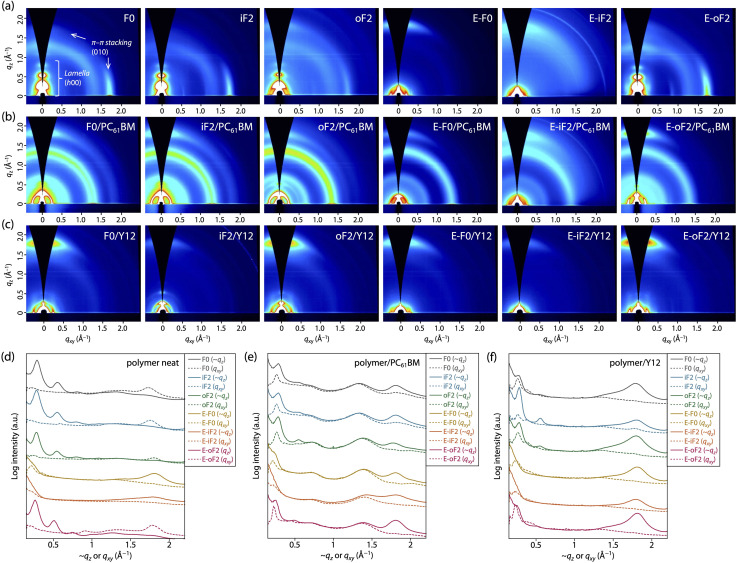
(a–c) 2D GIXD patterns and (d–f) cross-sectional profiles cut along the quasi-*q*_*z*_ axis and the *q*_*xy*_ axis for (a and d) polymer neat films, (b and e) polymer/PC_61_BM blend films, and (c and f) polymer/Y12 blend films.

We performed grazing incidence X-ray diffraction (GIXD) measurements to determine polymer order in the thin film. Fig. 6a and d display the two-dimensional (2D) GIXD patterns and the cross-sectional profiles cut from the 2D GIXD patterns for the polymer neat films. F0 provided a series of diffractions that were assignable to the lamellar order (*h* 0 0) up to the fourth order along the quasi-*q*_*z*_ (∼*q*_*z*_) axis and a diffraction assignable to the π–π stacking order (0 1 0) along the *q*_*xy*_ axis, indicative of an edge-on orientation.^10,47^ The *d*-spacing (*d*_π_) and the coherence length (*L*_π_) for the π–π stacking order were 3.53 Å and 33 Å, respectively (Table 3). iF2 and oF2 also showed similar 2D GIXD patterns, although oF2 showed some fraction of a π–π stacking diffraction along the ∼*q*_*z*_ axis, which was assignable to the face-on orientation.^10,47^ Whereas iF2 exhibited a *d*_π_ of 3.53 Å and an *L*_π_ of 34 Å, which were almost the same as those for F0, oF2 exhibited a slightly wider *d*_π_ of 3.58 Å and a slightly shorter *L*_π_ of 26 Å (Table 3). The slightly reduced polymer order for oF2 compared with those for F0 and iF2 could be due to the low molecular weight of oF2.

**Table 3:** π–π stacking distances and crystallite coherence lengths

Polymer (linkage models)	Acceptor	Edge-on π–π stacking		Face-on π–π stacking	
		*d*_π_ a (Å)	*L*_π_ b (Å)	*d*_π_ c (Å)	*L*_π_ b (Å)
F0 (R–H/H–H)	—	3.53	33	—	—
	PC_61_BM	—	—	3.49	28
	Y12	—	—	3.51	29
iF2 (R–H/F–F)	—	3.53	34	—	—
	PC_61_BM	—	—	3.49	27
	Y12	—	—	3.48	23
oF2 (R–F/H–H)	—	3.58	26	3.56	16
	PC_61_BM	—	—	3.57	22
	Y12	—	—	3.50	31
E-F0 (E–H/H–H)	—	—	—	3.49	29
	PC_61_BM	—	—	3.49	29
	Y12	—	—	3.48	25
E-iF2 (E–H/F–F)	—	—	—	3.51	14
	PC_61_BM	—	—	3.47	14
	Y12	—	—	3.52	24
E-oF2 (E–F/H–H)	—	3.53	35	—	—
	PC_61_BM	—	—	3.53	33
	Y12	—	—	3.47	35

a *d*-Spacing corresponds to the π–π structure of the edge-on fraction, (010) along the *q*_*xy*_ axis.

b Crystallite coherence length estimated from Scherrer's equation (*L*_π_ = 2π/FWHM) for the π–π stacking diffraction, where FWHM is the full width at half-maximum of the diffraction peak.

c *d*-Spacing corresponds to the π–π stacking of the face-on crystallite, (010) along the ≈*q*_*z*_ axis.

In contrast, the ester-substituted polymers (E-F0, E-iF2, and E-oF2) showed a different trend in the polymer order. E-F0 and E-iF2 exhibited diffraction patterns assignable to the face-on orientation, as evidenced by the fact that the π–π stacking diffraction was observed around the ∼*q*_*z*_ axis. Although both polymers had similar *d*_π_ values of around 3.5 Å, E-iF2 showed a significantly shorter *L*_π_ (14 Å) than E-F0 (29 Å) (Table 3). Although knowing that the diffraction along the *q*_*z*_ axis is not true data, we here compare *d*_π_ and *L*_π_ values determined from (0 1 0) diffraction along the *q*_*z*_ axis since they were obtained under the same conditions. On the other hand, E-oF2 exhibited a diffraction pattern that was assignable to the edge-on orientation with a *d*_π_ of 3.53 Å and an *L*_π_ of 35 Å (Table 3), similar to the alkyl-substituted polymers. As regards the face-on orientation observed for E-F0 and E-iF2, it is possible that the alkoxy moiety in the ester group can more freely rotate than the alkyl group, which diminishes the interchain interaction, giving rise to the alteration of the orientation from edge-on to face-on. The shorter *L*_π_ for E-iF2 than that for E-F0 could be ascribed to the significantly lower solubility of the former, which makes the polymer chains quickly aggregate and solidify, in turn preventing crystallization. In E-oF2, although it also had ester groups, the fluorine atom at the outer *β*-positions would play the role of a conformational directing group as discussed above, so that the E–F linkage highly preferred the *anti*-conformation, making the backbone more ordered. Thus, in E-oF2, the more ordered backbone may compensate for the effect of the alkoxy moiety, giving rise to the higher packing order and thereby an edge-on orientation.

### Polymer order in blend films

We also studied polymer order in the blend films, which were fabricated under the optimized conditions for each OPV cell, by 2D GIXD measurements. In the polymer/PC_61_BM blend films (Fig. 6b and e), all the polymers mainly adopted the face-on orientation as they exhibited the π–π stacking diffraction predominantly along the ∼*q*_*z*_ axis. Therefore, the orientation of F0, iF2, oF2, and E-oF2 was altered by blending with PC_61_BM. This was consistent with our previous studies.^34,40,41^ A plausible reason for the change in the backbone orientation is that the polymer chains can π–π interact with PC_61_BM molecules not only from the side but also from the top/bottom of the spherical fullerene moieties, forming the edge-on orientation and the face-on orientation, respectively.^10^ The *d*_π_ and *L*_π_ values were almost unchanged, though the orientation was changed, indicating that the polymer order was retained even by blending with PC_61_BM for all the polymers (Table 3). Consistent with the case of the neat films, *L*_π_ for E-iF2 was significantly short compared with the other polymers. Again, this is probably due to the significantly low solubility, preventing crystal growth.

In the polymer/Y12 blend films (Fig. 6c and f), the (0 1 0) π–π stacking diffraction also appeared along the ∼*q*_*z*_ axis in all cases. However, this included the π–π stacking diffractions of both polymer and Y12, which showed a face-on orientation (Fig. S6†). To probe the polymer order further in the polymer/Y12 blend films, we focused on the (1 0 0) lamellar diffraction for the polymer that appeared in the small-angle region. F0/Y12 and oF2/Y12 blend films exhibited a clear lamellar diffraction in the *q*_*xy*_ axis, consistent with the clear π–π stacking diffraction, meaning that F0 and oF2 indeed formed crystalline structures in the Y12 blend film. On the other hand, the lamellar diffraction for the iF2/Y12 blend film was relatively weak, consistent with the weak π–π stacking diffraction. Such a relationship between the π–π stacking and lamellar diffractions in the polymer/Y12 blend film was also seen in the ester-substituted polymers. Therefore, we can safely compare the polymer crystallinity in the polymer/Y12 blend film by using the *L*_π_ values. Notably, the 2D GIXD images mean that the orientation of F0, iF2, oF2, and E-oF2 was also altered by blending with Y12. This is probably because the orientation of the polymers was affected by that of Y12 (Fig. S6†). F0 and oF2 showed longer *L*_π_ values (29 Å and 35 Å) than iF2 (24 Å) (Table 3). Interestingly, the fact that oF2 showed a longer *L*_π_ value than iF2 contrasted with the fact that iF2 showed a longer *L*_π_ value than oF2 in the PC_61_BM blend film. Seemingly, the higher solubility in oF2 than in iF2 was beneficial to form a higher crystalline structure particularly in the Y12 blend. In the ester-substituted polymers, E-oF2 showed a longer *L*_π_ value (35 Å) than E-F0 (25 Å) and E-iF2 (24 Å) (Table 3). Similarly, E-oF2 with higher solubility had higher crystallinity than E-iF2.

Overall, in the PC_61_BM blend film, the trend of crystallinity was similar to that in the neat film. On the other hand, in the Y12 blend film, the polymers having higher solubility tended to show higher crystallinity. It is likely that the difference in the aggregation properties between PC_61_BM and Y12 affects the growth of the polymer crystallites during the drying process.

### Blend morphology

**Fig. 7:**
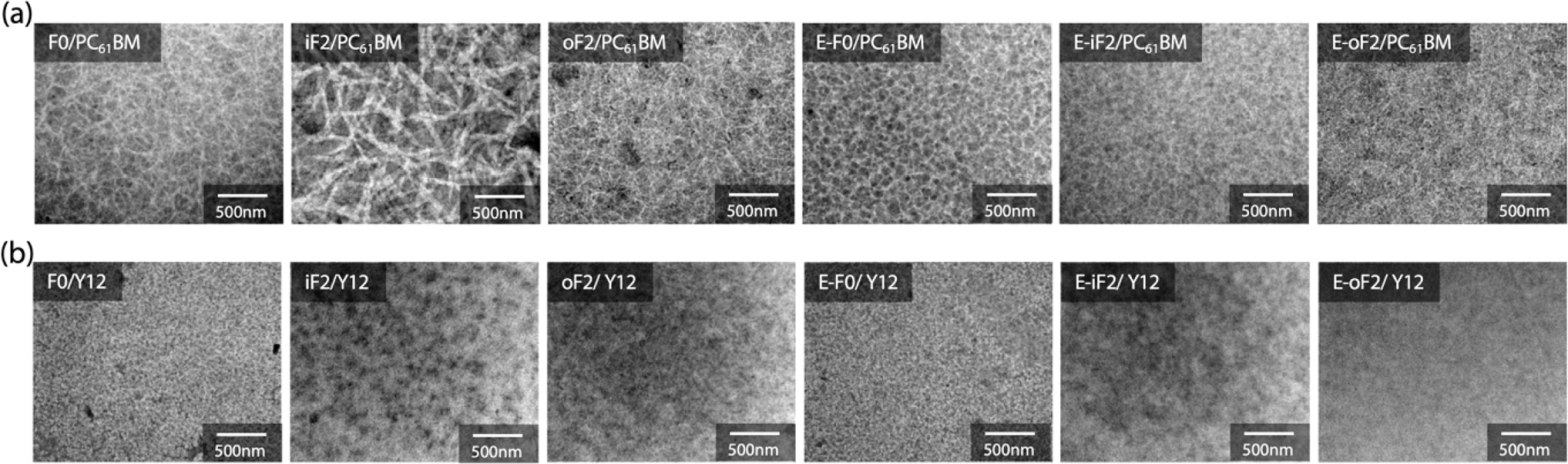
TEM images of (a) polymer/PC_61_BM and (b) polymer/Y12 blend films.

The morphology of the blend films was investigated by transmission electron microscopy (TEM). Fig. 7a and b depict the TEM images of the polymer/PC_61_BM and polymer/Y12 blend films that were fabricated under the optimized conditions for each OPV cell.

In the polymer/PC_61_BM blend films, we observed fibrillar network structures for all the blends, which were likely formed by the polymer aggregates. For the alkyl-substituted polymers, iF2 exhibited thicker fibrils than F0 and oF2, presumably because of the higher aggregation properties of iF2 than the other polymers. On the other hand, all ester-substituted polymers had similar network structures. We note that even though E-iF2 had higher aggregation properties similar to iF2, fibril width in the E-iF2 blend film was smaller than that in the iF2 blend film, probably because E-iF2 showed much lower crystallinity than iF2 in the PC_61_BM blend film.

In the polymer/Y12 blend films, we did not observe such clear fibrillar network structures observed in the polymer/PC_61_BM blend films. This implies that the polymers were more mixed and formed a more finely phase-separated structure with Y12 than with PC_61_BM. This is consistent with the fact that the difference in interfacial energy between the polymer and the acceptor was smaller in the polymer/Y12 blend than in the polymer/PC_61_BM blend (Fig. S7 and Table S2†) and therefore, the former was more miscible.^48^ Nevertheless, iF2 and E-iF2 seemed to have formed somewhat large phase separation compared with the other polymers, which might be due to their high aggregation properies.

### Photovoltaic performance

**Fig. 8:**
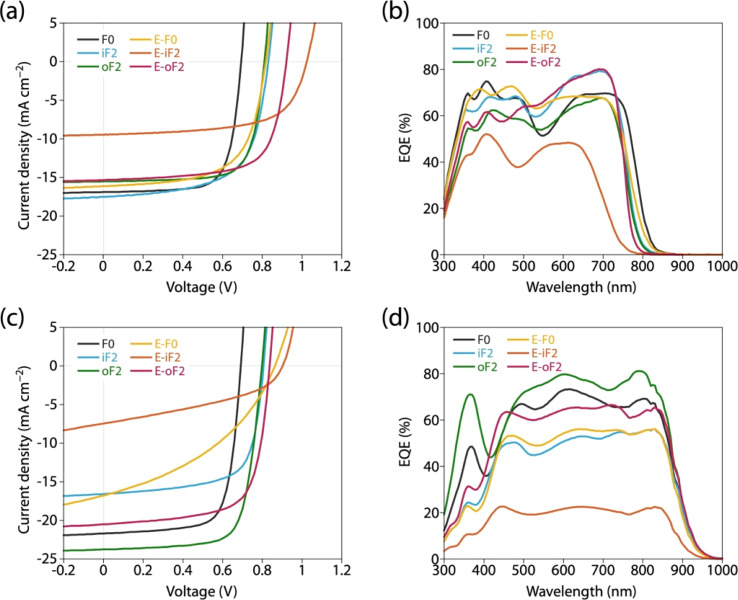
(a and c) *J*–*V* curves and (b and d) EQE spectra of photovoltaic cells. (a and b) PC_61_BM-based cells and (c and d) Y12-based cells.

We fabricated photovoltaic cells with an inverted structure (ITO/ZnO/polymer:acceptor/MoO_*x*_/Ag), where the polymers were used as the donor and PC_61_BM and Y12 were used as the acceptors. For all the cells, the photoactive layer was prepared by spin-coating the blend solution. Fig. 8a and c depict the current–voltage (*J*–*V*) curves and Fig. 8b and d depict the external quantum efficiency (EQE) spectra of the cells. Table 4 summarizes the photovoltaic properties of the cells along with the polymer physical features, blend structural features, and PL quenching efficiency. For all the cells, the *J*_SC_ value obtained by *J*–*V* measurement was consistent with the *J*_SC_ value integrated from the EQE spectrum (*J*^EQE^_SC_).

**Table 4:** Summary of polymer properties and blend structure and properties, and photovoltaic properties

Polymer (linkage models)	Solubility at 100 °C	Aggregation	Crystallinity in the blend	Morphology in the blend	PL quenching^a^ (%)	Acceptor	*J*_SC_ (*J*^EQE^_SC_)^b^ (mA cm^−2^)	*V*_OC_ (V)	FF (−)	PCE (PCE_ave_)^c^ (%)
F0 (R–H/H–H)	High	High	High	Good	96	PC_61_BM	16.9 (16.5)	0.69	0.73	8.6 (8.3)
			High	Good	95	Y12	21.7 (21.5)	0.69	0.73	10.9 (10.6)
iF2 (R–H/F–F)	Low	Very high	High	Fair	97	PC_61_BM	17.5 (17.3)	0.83	0.64	9.3 (9.0)
			Moderate	Fair	94	Y12	16.6 (16.5)	0.81	0.69	9.2 (8.9)
oF2 (R–F/H–H)	High	Moderate	Moderate	Good	97	PC_61_BM	15.9 (15.6)	0.82	0.75	9.7 (9.3)
			High	Good	94	Y12	24.0 (23.7)	0.79	0.72	13.9 (13.4)
E-F0 (E–H/H–H)	High	High	High	Good	99	PC_61_BM	16.1 (15.8)	0.82	0.63	8.4 (8.0)
			Moderate	Good	93	Y12	16.8 (16.5)	0.86	0.40	5.8 (5.3)
E-iF2 (E–H/F–F)	Low	Very high	Low	Good	99	PC_61_BM	9.4 (9.2)	1.02	0.63	6.1 (5.8)
			Low	Fair	31	Y12	7.5 (7.3)	0.90	0.40	2.7 (2.4)
E-oF2 (E–F/H–H)	High	High	High	Good	96	PC_61_BM	15.3 (15.0)	0.92	0.69	9.8 (9.4)
			High	Good	70	Y12	20.5 (20.2)	0.84	0.70	11.9 (11.6)

a For the PC_61_BM blends, the film was excited at 600 nm (polymers were selectively excited). For the Y12 blends, the film was excited at 820 nm (Y12 was selectively excited).

b *J* ^EQE^_SC_: *J*_SC_ calculated from the EQE spectrum.

c PCE: maximum PCE. PCE_ave_: average PCE from more than 10 cells (40 active areas).

**Fig. 9:**
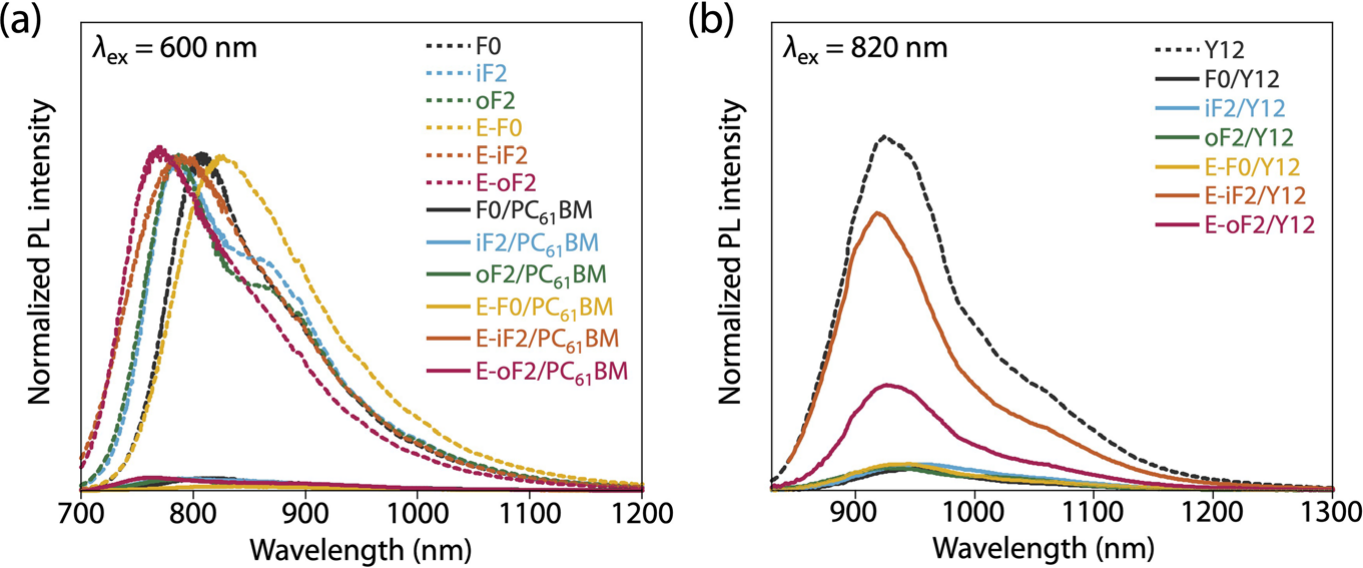
Photoluminescence spectra of (a) polymer thin films and polymer/PC_61_BM blend films (excited at 600 nm) and (b) Y12 neat film and polymer/Y12 blend films (excited at 820 nm).

In all the polymer/PC_61_BM-based cells, the optimal polymer-to-PC_61_BM weight ratio was 1 : 2. F0 and iF2 showed a PCE value of 8.6% (*J*_SC_ = 16.9 mA cm^−2^, *V*_OC_ = 0.69 V, fill factor (FF) = 0.73) and 9.3% (*J*_SC_ = 17.5 mA cm^−2^, *V*_OC_ = 0.83 V, FF = 0.64), respectively, consistent with our previous reports.^40^ Note that the lower FF of the iF2 cell than that of the F0 cell in spite of the relatively high mobility is due to the enhanced bimolecular recombination.^40^ The higher *V*_OC_ for iF2 than that for F0 corresponded to the deeper *E*_HOMO_. The low FF for iF2 could be ascribed to the increased recombination owing to the deteriorated morphology originating in the low solubility. The oF2 cell showed a slightly lower *J*_SC_ and a considerably higher FF than the iF2 cell, resulting in a similar PCE of 9.7% (*J*_SC_ = 15.9 mA cm^−2^, *V*_OC_ = 0.82 V, FF = 0.75). The photoluminescence (PL) quenching study revealed that F0, iF2, and oF2 showed similarly high PL quenching efficiencies of more than 95% when the polymers were excited (*λ*_ex_ = 600 nm) (Fig. 9a and Table 4), which agrees well with the sufficiently large Δ*E*_L_ values (Table 2), and thus the electron transfer from the polymer to PC_61_BM was evenly efficient. Therefore, the slightly lower *J*_SC_ in oF2 than in iF2 could be due to the slightly lower crystallinity, which possibly originates from the relatively low molecular weight of oF2. On the other hand, the high FF in oF2 could be ascribed to the finely phase-separated morphology perhaps owing to the increased solubility compared to iF2, thereby avoiding charge recombination.

E-F0 showed a *J*_SC_ of 16.1 mA cm^−2^, which was similar to that for F0 (16.9 mA cm^−2^). This was quite reasonable that the crystallinity and morphology of these polymers were similar. The *V*_OC_ for E-F0 (0.82 V) was significantly increased from that for F0 (0.69 V) due to the deeper *E*_HOMO_ originating from the replacement of the alkyl groups with the ester groups. However, E-F0 showed an FF of 0.63 which was lower than F0 (0.73), resulting in a PCE of 8.4% which was similar to that of F0 (8.6%). E-F0 and E-iF2 showed lower FF values (both 0.63), which was consistent with the lower charge carrier mobility than the others (Fig. S8 and Table S3†). E-iF2 provided a low *J*_SC_ of 9.4 mA cm^−2^. As the PL quenching efficiency for E-iF2 in the PC_61_BM blend was as high as 99%, which was similar to the other polymers and was consistent with Δ*E*_L_, the low *J*_SC_ can be ascribed to the low crystallinity. As a result, although the *V*_OC_ was as high as 1.02 V, reflecting the deepest *E*_HOMO_ among the present polymers, E-iF2 showed a lower PCE of 6.1%. By contrast, E-oF2 showed a *J*_SC_ of 15.3 mA cm^−2^ and a *V*_OC_ of 0.92 V, resulting in the highest PCE of 9.8% among the present polymers. These *J*_SC_ and *V*_OC_ values were similar to and higher than those of E-F0 (without fluorine substitution) and oF2 (alkyl counterpart), which were consistent with the crystallinity and *E*_HOMO_.

In the Y12-based cells, F0 exhibited a PCE of 10.9%, a *J*_SC_ of 21.7 mA cm^−2^, a *V*_OC_ of 0.69 V, and an FF of 0.73. In contrast to the PC_61_BM-based cell, iF2 showed a lower *J*_SC_ and thereby a lower PCE than F0 in the Y12-based cell; PCE of the iF2/Y12 cell was 9.2% (*J*_SC_ = 16.6 mA cm^−2^, *V*_OC_ = 0.81 V, FF = 0.69). This can be ascribed to the fact that iF2 showed lower crystallinity than F0 in the Y12 blend. With a similar crystallinity to and a deeper *E*_HOMO_ than F0, oF2 had a higher PCE of 13.9% (*J*_SC_ = 24.0 mA cm^−2^, *V*_OC_ = 0.79 V, FF = 0.72), the highest among the polymers studied here. For E-F0 and E-iF2, although their *V*_OC_ values were higher than those of the other polymers, reflecting their deep *E*_HOMO_s, *J*_SC_ and FF were considerably lower, resulting in low PCEs of around 3–6%. This was also consistent with the lower crystallinity of E-F0 and E-iF2. In addition, because the PL quenching efficiency for the E-iF2/Y12 blend films when excited at Y12 absorption wavelength (*λ*_ex_ = 820 nm) was limited to approximately 30%, originating from the relatively small Δ*E*_H_ value (Table 2), whereas the quenching efficiencies for the blends of E-F0 and the alkylated polymers were higher than 90% (Fig. 9b and Table 4), the low *J*_SC_ for the E-iF2/Y12 cell compared to the other Y12 cells was also ascribed to the inefficient hole transfer from Y12 to the polymer. For E-oF2, although the PL quenching efficiency was also low (approx. 70%) because of the smaller Δ*E*_H_ (Table 2), the *J*_SC_ value was relatively high (20.5 mA cm^−2^), most likely owing to the high crystallinity. As a result, the E-oF2 cell gave a relatively high PCE of 11.9% with a *V*_OC_ of 0.84 V and an FF of 0.70.

To summarize, the variation of *V*_OC_ was mostly consistent with the difference in the *E*_HOMO_ of the polymers in both the cells based on PC_61_BM and Y12. The *J*_SC_ mostly followed the crystallinity in the blend film: the smaller Δ*E*_H_ also affected the Y12-based cell. The FF was determined by the combination of crystallinity and morphology in the blend film: although E-F0 was an exception, its lower mobility agreed well.

## Conclusions

We designed and synthesized a series of NTz-based polymers by systematically changing the combination of the side chain (alkyl or ester group) and the fluorination position in the backbone. By doing so, the locations of the intramolecular noncovalent interactions, namely, O⋯S and F⋯S interactions, in these polymers were systematically changed. The energy levels of the polymers lowered by reflecting the electronic effects of the ester and fluorine groups. As the ester and fluorine groups were introduced in the positions where the HOMO density was relatively high, the shift of the HOMO energy level was more significant than that of the LUMO energy level, which correlated well with the variation of the optical bandgap.

The solubility, aggregation properties, and crystallinity of the polymers varied significantly, which was well explained by the effects of these noncovalent interactions. In fact, replacing the alkyl group with the ester group did not significantly lower the solubility of the polymer although it induces an O⋯S interaction. When two fluorine groups were introduced at the inner *β*-positions of the bithiophene moiety, bithiophene is strongly interlocked by the two F⋯S interactions, leading to a highly rigid backbone and strong aggregation and thereby significantly decreased solubility. In contrast, when fluorine groups were introduced at the outer *β*-positions of the bithiophene moiety, these fluorine groups induce a weaker F⋯S interaction with the alkylthiophene or esterthiophene attached to the bithiophene moiety and act as the conformation-directing group, leading to a more flexible backbone and weaker aggregation but a similarly ordered backbone. We also found that the polymer solubility is a more important factor to have high crystallinity in the nonfullerene blend than in the fullerene blend. As a result, the photovoltaic performance of the polymers in both fullerene- and nonfullerene-based cells correlated well with the energetics, physical properties, and structural orders of the polymers.

To conclude, this study clearly shows that the introduction of the electron-withdrawing groups that can induce noncovalent intramolecular interactions is a powerful strategy to create the π-conjugated polymers with lower HOMO energy levels and high structural orders, which is crucial for improving the photovoltaic performance, as has been studied so far. More importantly, we found that the careful design of the introduction positions of those functional groups can successfully afford such polymers without sacrificing the solubility that is essential for solution-processing and is, found to be, crucial for forming higher crystallinity particularly in the nonfullerene blend. We believe that our systematic study offers important hints for the design of π-conjugated polymers for efficient OPVs.

## Data availability

ESI† is available and includes the synthesis and characterization of the compounds, experimental procedures, GPC charts, DSC curves, PYS and LEIPS data, HOMO and LUMO geometries, contact angle data, and CV and 2D GIXD for the acceptors.

## Author contributions

S. K. and M. S. contributed equally to this work. S. K. synthesized all the polymers. S. K., M. S., Y. T., and K. Y. conducted fabrication and measurements of OPV cells. S. K. and M. S. carried out the UV-vis, CV, PYS, DFT calculations, and 2D GIXD experiments. H. I., A. S., and H. Y. conducted LEIPS measurements. J. J, H. D. K., and H. O. carried out PL measurements. S. K., M. S., H. O., and I. O. prepared the manuscript, and all authors discussed and commented on the manuscript. I. O. directed the project.

## Conflicts of interest

There are no conflicts to declare.

## Supplementary Material

SC-015-D4SC00899E-s001
