# Supplementary material for: Manipulating the functionality and structures of π-conjugated polymers utilizing intramolecular noncovalent interactions towards efficient organic photovoltaics
Source: Chem Sci. 2024 Mar 25;15(17):6349–62. doi: 10.1039/d4sc00899e (PMC11062120; doi:10.1039/d4sc00899e)
Supplement: SC-015-D4SC00899E-s001 [file SC-015-D4SC00899E-s001.pdf]

Electronic Supplementary Material (ESI) for Chemical Science.
This journal is © The Royal Society of Chemistry 2024

Supporting Information

Manipulating Functionality and Structures of π-Conjugated Polymers Utilizing Intramolecular Noncovalent Interactions Towards Efficient Organic Photovoltaics

Satoshi Kamimura,[1] Masahiko Saito,[1,2] Yoshikazu Teshima,[2] Kodai Yamanaka,[2] Hiroyuki Ichikawa,[3] Ai Sugie,[3] Hiroyuki Yoshida,[3,4] Jihun Jeon,[5] Hyung Do Kim,[5] Hideo Ohkita,[5] Tsubasa Mikie, [1,2] and Itaru Osaka[1,2,]*

[1]Department of Applied Chemistry, Graduate School of Engineering, Hiroshima University, Higashi-Hiroshima, Hiroshima 739-8527

[2]Applied Chemistry Program, Graduate School of Advanced Science and Engineering, Hiroshima University, Higashi-Hiroshima, Hiroshima 739-8527

[3]Department of Materials Science, Graduate School of Engineering, Chiba University 1-33 Yayoi-cho Inage-ku, Chiba 263-8522, Japan

[4]Molecular Chirality Research Center, Chiba University 1-33 Yayoi-cho Inage-ku, Chiba 263-8522, Japan

[5]Department of Polymer Chemistry, Graduate School of Engineering, Kyoto University, Katura, Nishikyo-ku, Kyoto 615-8510, Japan

Corresponding author e-mail: iosaka@hiroshima-u.ac.jp

1. Materials and synthesis

All chemicals were used as purchased. $PC_{61}BM$ and Y12 were purchased from Solenne BV and Ossila Ltd., respectively. 5,10-Bis(5-bromo-4-(2-dodecylhexadecyl)thiophene-2-yl)-naphtho[1,2-*c*:5,6-*c*′]bis[1,2,5]thiadiazole (**1**),[S1] **PNTz4T** (**F0**), **PNTz4TF2** (**iF2**),[S2] 5,10-bis(4,4,5,5-tetramethyl-1,3,2-dioxaborolan-2-yl)naphtho[1,2,-*c*:5,6,-*c*′]bis([1,2,5]thiadiazole) (**NTz-Bpin2**),[S3] 5,5′-bis(trimethylstannyl)-2,2′-bithiophene (**2**),[S4] 5,5′-bis(trimethylstannyl)-3,3′-difluoro-2,2′-bithiophene (**3**)[S2] and 5,5′-bis(trimethylstannyl)-4,4′-difluoro-2,2′-bithiophene (**4**)[S5] were synthesized according to the reported procedures. Nuclear magnetic resonance (NMR) spectra were taken on a Varian-400 spectrometer or a Varian-500 spectrometer, using $CDCl_3$ calibrated with the chloroform peak at 7.26 ppm for ^{1}H-NMR spectra and at 77.2 ppm for ^{13}C-NMR spectra, respectively. High-resolution mass spectroscopy (HRMS) was performed using LTQ Orbitrap XL (Thermo Fisher Scientific, Inc.). Molecular weights were determined by high-temperature gel permeation chromatography (GPC), using TOSOH HLC-8321GPC/HT at 140 °C with *o*-dichlorobenzene as the solvent, which was calibrated with polystyrene standards.

Synthesis of 2-bromothiophene-3-carboxylic acid (6)

Lithium diisopropyl amide (LDA), which was prepared from diisopropylamine (5.5 mL, 39.0 mmol) and 1.57 M *n*-butyllithium (25 mL, 39.0 mmol) in tetrahydrofuran (THF) (60 mL), was added dropwise to a THF (60 mL) solution of 3-thiophenecarboxylic acid (**5**) (2.00 g, 15.6 mmol) at −30 °C. After the mixture was stirred for 1 h, tetrabromomethane (12.9 g, 39.0 mmol) was added in one portion at −30 °C. After stirring for another 1 h, the mixture was warmed to room temperature. Dilute hydrochloric acid was added to the mixture, which was extracted with ether three times. The organic layer was dried over magnesium sulfate, and the solvent was evaporated under a reduced pressure. The residue was purified by recrystallization from ethanol:water (1:4) to give **6** as a white solid (5.2 g, 64%). ^{1}H-NMR (400 MHz, $CDCl_3$) δ: 7.44

(d, J = 5.1 Hz, 1H), 7.27 (d, J = 5.1 Hz, 1H).

Synthesis of 2-bromo-5-iodothiophene-3-carboxylic acid (7)

Under argon atmosphere, **6** (3.33 g, 16.1 mmol), iodine (3.26 g, 12.9 mmol), and periodic acid dihydrate (1.10 g, 4.82 mmol) were added in a mixed solvent of acetic acid and water (2:1 v/v, 180 mL), which was heated to 90 °C. After stirring for 9 h, the reaction mixture was cooled to room temperature, and sodium thiosulfate was added. The resulting precipitate was filtered and washed with water. The residue was recrystallized from chloroform to give **7** as a white crystal (4.04 g, 75%). ^{1}H-NMR (400 MHz, $CDCl_3$) δ: 7.59 (s, 1H). ^{13}C-NMR (101 MHz, $CDCl_3$) δ: 166.11, 143.66, 134.23, 123.20, 69.24. HRMS calcd for $C_5H_2BrIO_2S$ $[M-H]^-$: 331.8004. Found: 330.7933.

Synthesis of 2-decyltetradecyl 2-bromo-5-iodothiophene-3-carboxyrate (8)

Under argon atmosphere, **7** (2.00 g, 6.01 mmol), 2-decyltetradecylbromide (3.76 g, 9.01 mmol), and potassium carbonate (16.61 g, 120 mmol) were added in DMF (80 mL), which was heated to 85 °C. After stirring for 12 h, the mixture was cooled to room temperature and water was added. The mixture was extracted with ethyl acetate for three times and the combined organic layer was washed with water for four times and dried over $MgSO_4$. After removal of the solvent by evaporation, the residue was purified by column chromatography on silica gel with hexane:dichloromethane gradient (1:0 to 3:1) to give **8** as a colorless oil (3.85g, 96%). ^{1}H-NMR (400 MHz, $CDCl_3$) δ: 7.52 (s, 1H), 4.18 (d, J = 4.2 Hz, 2H), 1.73 (m, 1H), 1.41–1.20 (m, 46H), 0.88 (m, 3H). ^{13}C-NMR (101 MHz, $CDCl_3$) δ: 160.68, 137.67, 132.31, 121.75, 68.01, 40.19, 37.85, 31.91, 31.31, 29.90, 29.68, 29.63, 29.58, 29.34, 26.71, 23.28, 13.25. HRMS calcd for $C_{29}H_{50}BrIO_2S$ $[M+H]^+$: 691.1760. Found: 691.1660.

Synthesis of bis(2-decyltetradecyl) 5,5′-(naphtho[1,2-*c*:5,6-*c*′]bis([1,2,5]thiadiazole)-5,10-diyl)bis(2-bromothiophene-3-carboxylate) (9)

Under argon atmosphere, **8**, **NTz-Bpin2** (496 mg, 1 mmol), $Pd(PPh_3)_4$ (103 mg, 0.09 mmol), and Aliquat 336 (3 drops) were added in toluene (40 mL), which was heated to 60 °C. After stirring for 30 min, K_2CO_3aq (829 mg in 15 mL of water) was added and heated 95 °C. After stirring for 20 h, the mixture was cooled to room temperature and extracted with toluene for three times. The combined organic layer was washed with water and dried over $MgSO_4$. After removal of the solvent by evaporation, the residue purified by column chromatography on silica gel with chloroform:hexane (3:1) followed by recrystallization from chloroform:ethanol (1:4) to give **9** as a red solid (486 mg, 37%). ^{1}H-NMR (400 MHz, $CDCl_3$) δ: 9.07 (s, 2H), 8.46 (s, 2H), 4.29 (d, J = 4.2 Hz, 4H), 1.84 (m, 2H), 1.41-1.20 (m, 92H), 0.85 (m, 6H). ^{13}C-NMR (101 MHz, $CDCl_3$) δ: 161.92, 152.84, 151.57, 138.21, 132.19, 129.21, 125.02, 124.67, 121.92, 68.18, 37.43, 31.91, 31.44, 30.05, 29.71, 29.67, 29.35, 26.80, 22.67, 14.09. HRMS calcd for $C_{68}H_{102}Br_2N_4O_4S_4$ $[M+H]^+$: 1325.5151. Found: 1325.5249.

Synthesis of oF2

To a reaction tube equipped with a stirring bar, **1** (62 mg, 0.05 mmol), **4** (26 mg, 0.05 mmol), $Pd(PPh_3)_4$ (1.2 mg, 0.001 mmol) and toluene (2 mL) were added. The tube was purged with argon and sealed. The tube was then set into a microwave reactor and heated at 200 °C for 2 h. After cooling to room temperature, the reaction mixture was poured into 50 mL of methanol containing 2 mL of hydrochloric acid, which was stirred for 1 h. Then the precipitate was collected by filtration and subjected to the sequential Soxhlet extraction with methanol, hexane and dichloromethane to remove low molecular weight fractions. The residue was then extracted with chloroform. The chloroform solution was concentrated and reprecipitated in methanol. The precipitated was collected by filtration and dried in vacuum to yield **oF2** as a dark green solid (48 mg, 75%, M_n = 33,800, M_w =65,800). Anal. Calcd for $C_{74}H_{106}F_2N_4S_6$: C, 69.33; H,

8.33, N, 4.37. Found: C, 67.88; H, 7.72, N, 4.22.

Synthesis of E-F0

To a reaction tube equipped with a stirring bar, **9** (66 mg, 0.05 mmol), **2** (26 mg, 0.05 mmol), $Pd(PPh_3)_4$ (1.2 mg, 0.001 mmol) and toluene (2 mL) were added. The tube was purged with argon and sealed. The tube was then set into a microwave reactor and heated at 180 °C for 1 h. After cooling to room temperature, the reaction mixture was poured into 50 mL of methanol. Then the precipitate was collected by filtration and subjected to the sequential Soxhlet extraction with methanol, hexane, dichloromethane and chloroform to remove low molecular weight fractions. The residue was then extracted with chlorobenzene. The extracted chlorobenzene solution was concentrated and reprecipitated in methanol. The precipitated was collected by filtration and dried in vacuum to yield **E-F0** as a dark green solid (61 mg, 92%, M_n = 124,000, M_w = 241,000). Anal. Calcd for $C_{76}H_{108}N_4O_4S_6$: C, 68.42; H, 8.16, N, 4.20. Found: C, 65.40; H, 7.85, N, 3.99.

Synthesis of E-iF2

E-iF2 was synthesized in the same way as **E-F0** by using **9** and **3** as the comonomers, yielding the polymer as a dark green solid (58 mg, 84%, M_n = 44,500, M_w = 100,000). Anal. Calcd for $C_{76}H_{106}F_2N_4O_4S_6$: C, 66.63; H, 7.80, N, 4.09. Found: C, 65.59; H, 7.67, N, 4.01.

Synthesis of E-oF2

E-oF2 was synthesized in the same way as **E-F0** by using **9** and **4** as the comonomers, yielding the polymer as a dark green solid (56mg, 82%, M_n = 43,800, M_w =96,600). Anal. Calcd for $C_{76}H_{108}N_4O_4S_6$: C, 66.63; H, 7.80, N, 4.09. Found: C, 65.23; H, 7.45, N, 4.03.

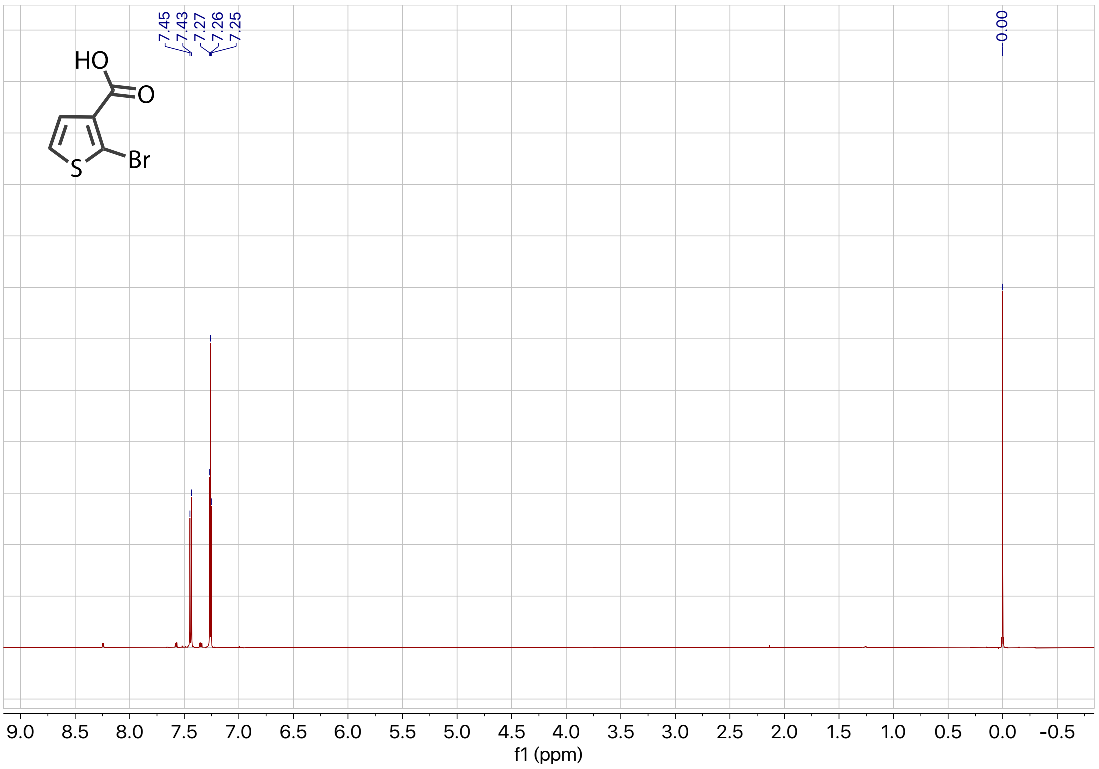

^{1}H-NMR spectra of **6**

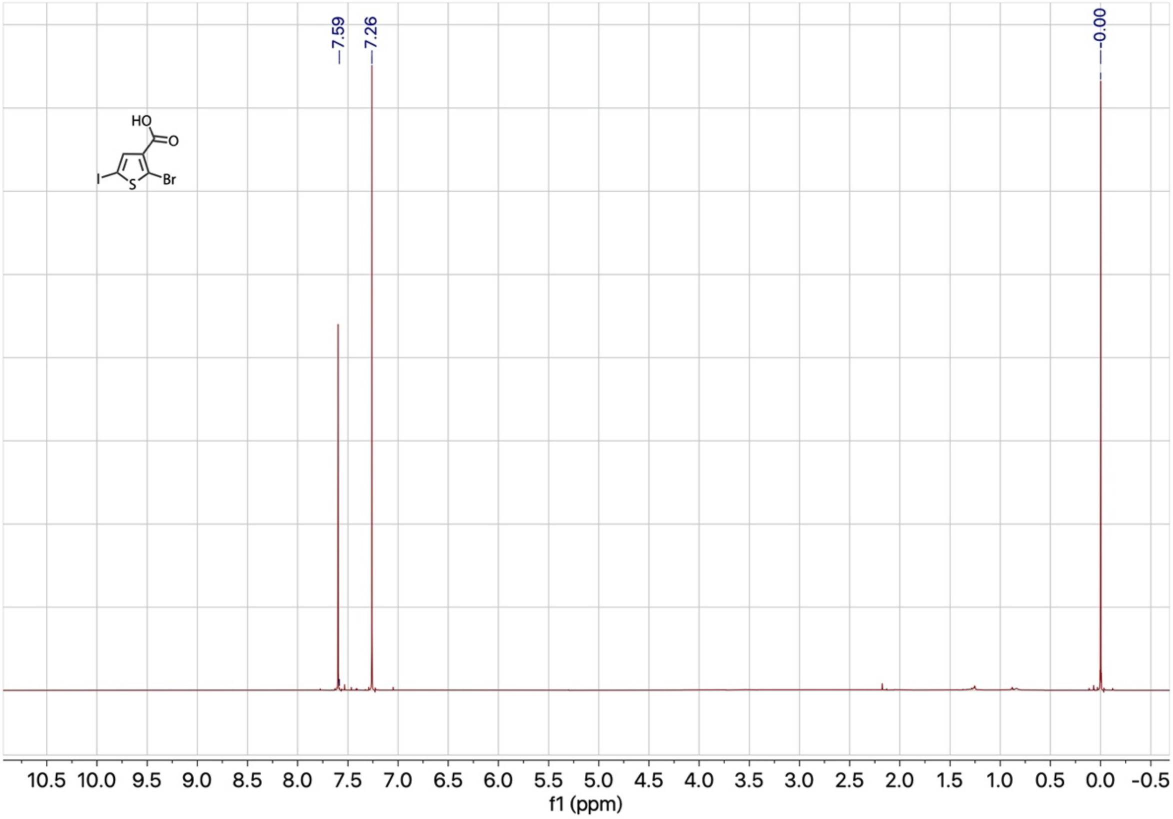

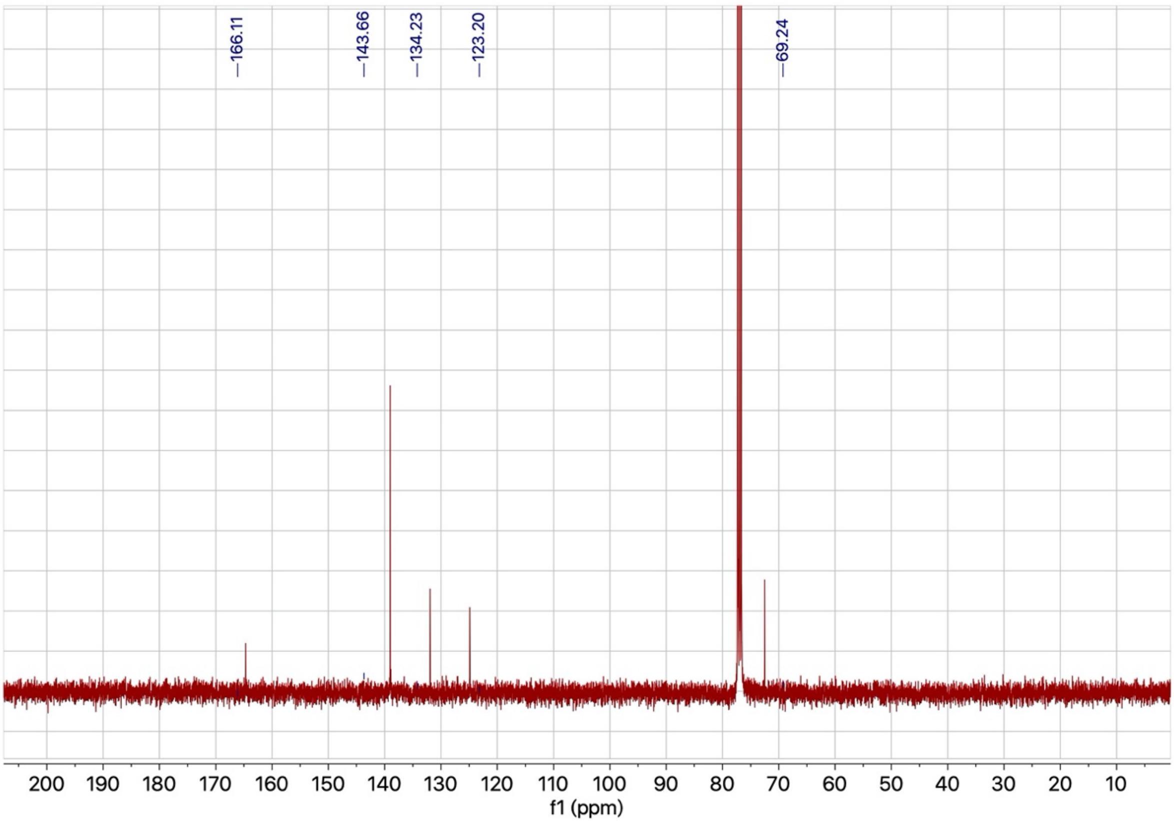

^{1}H- (upper) and ^{13}C- (lower) NMR spectra of **7**

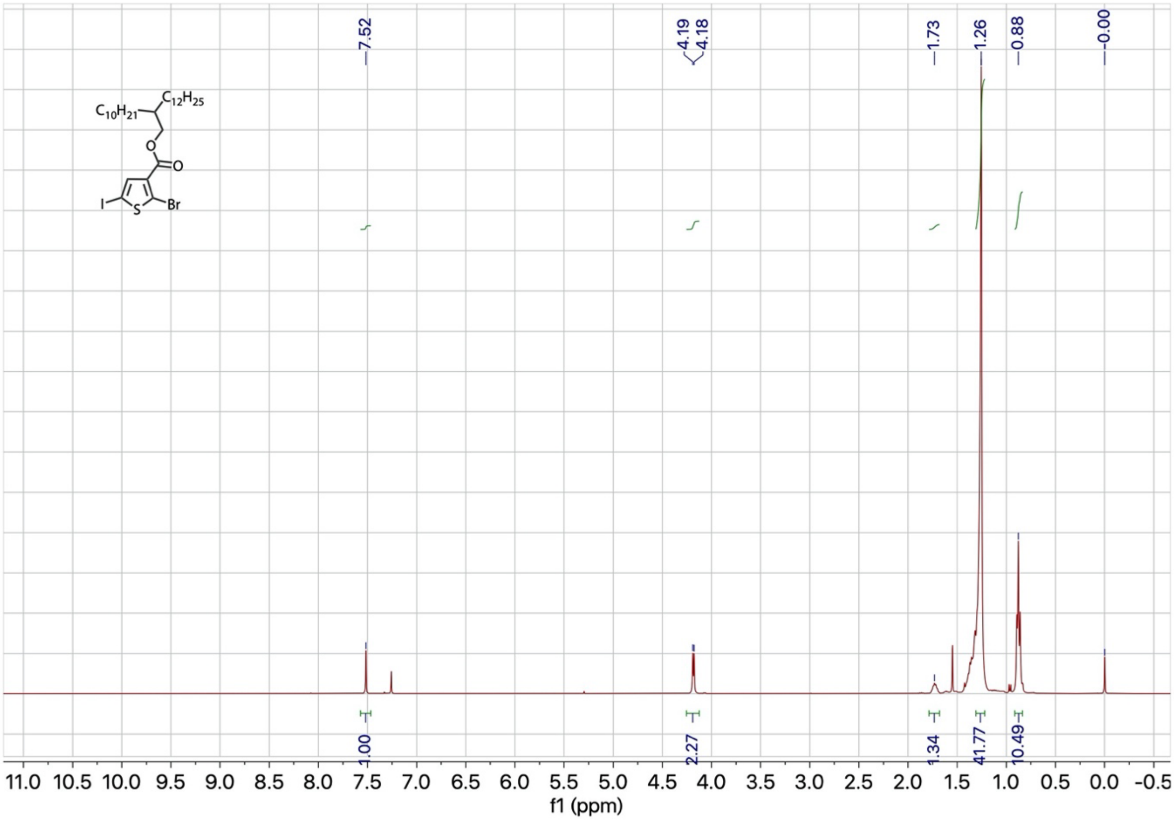

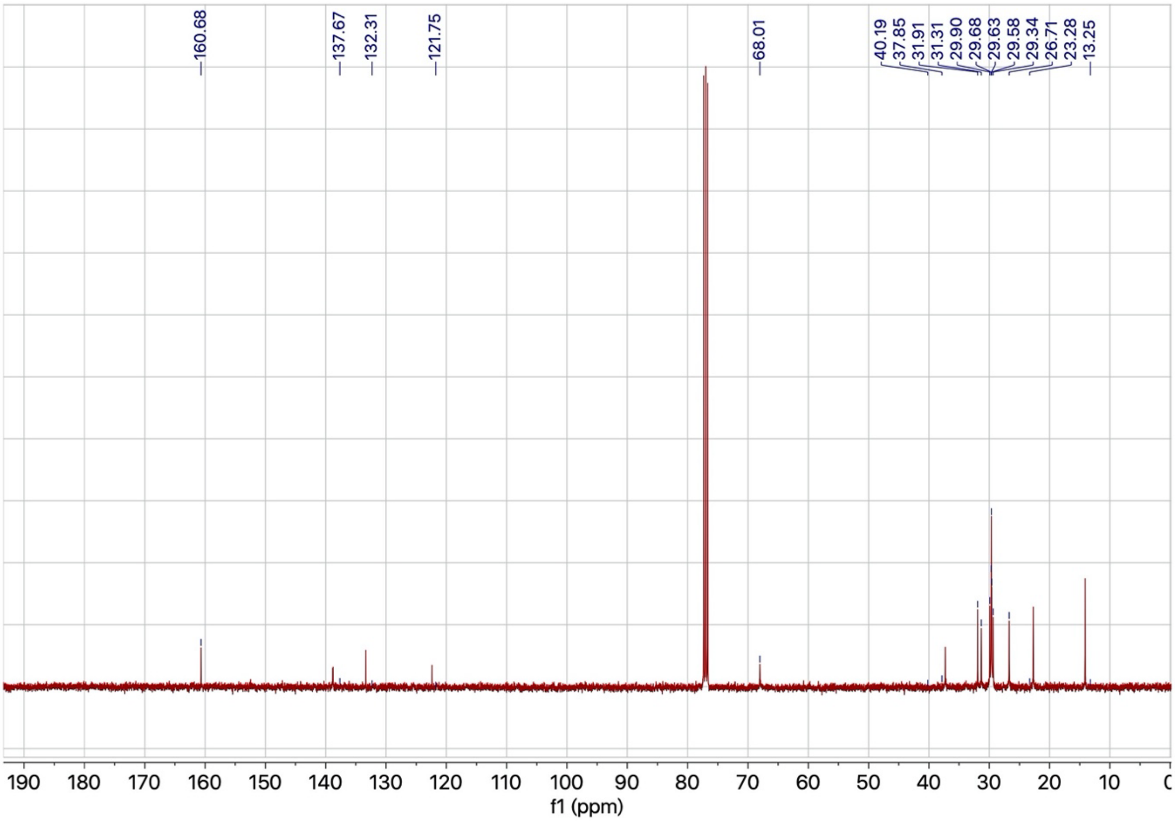

^{1}H- (upper) and ^{13}C- (lower) NMR spectra of **8**

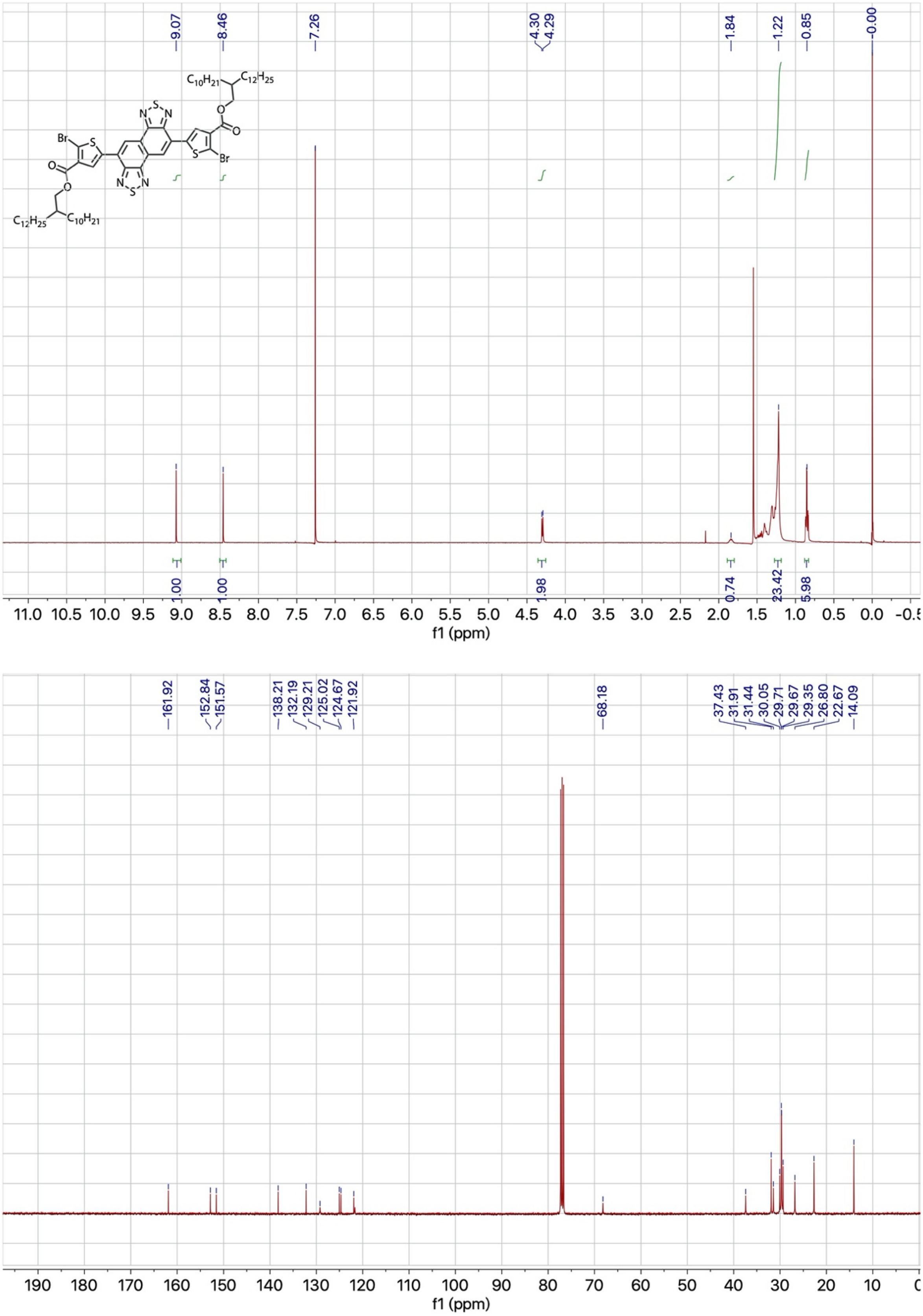

^{1}H- (upper) and ^{13}C- (lower) NMR spectra of **9**

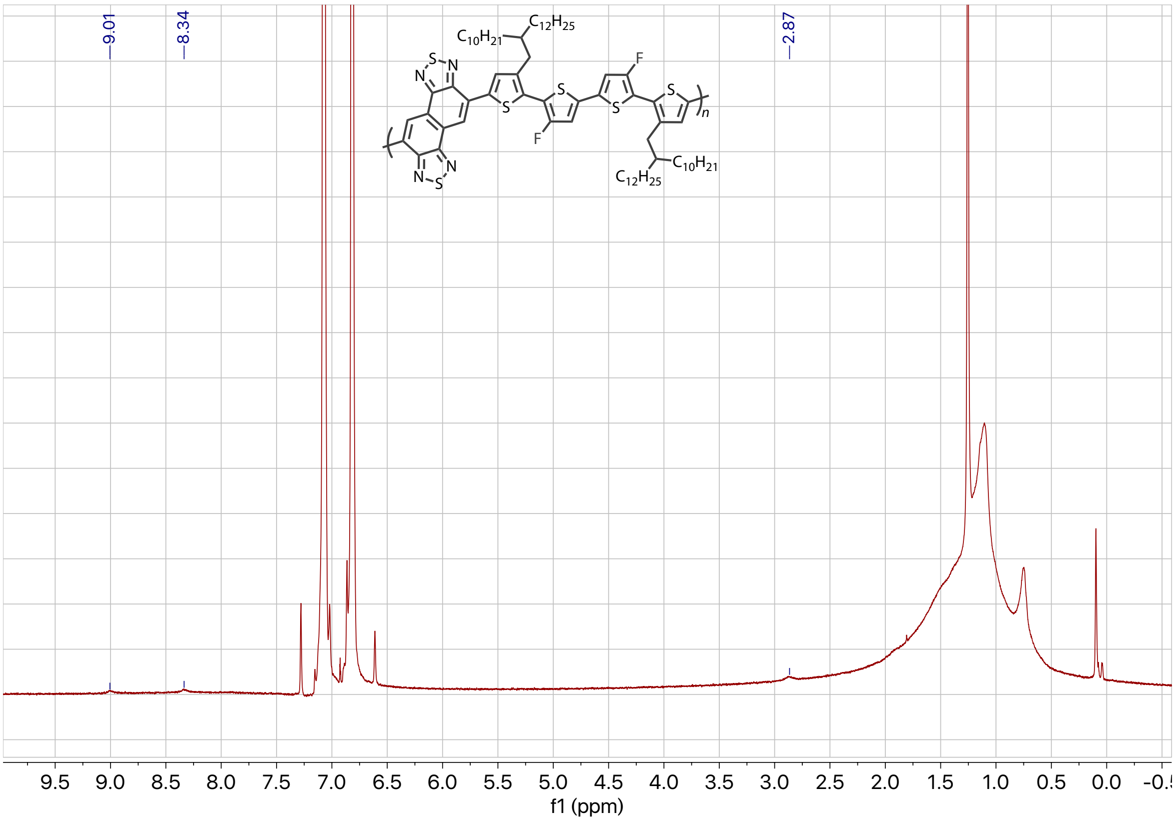

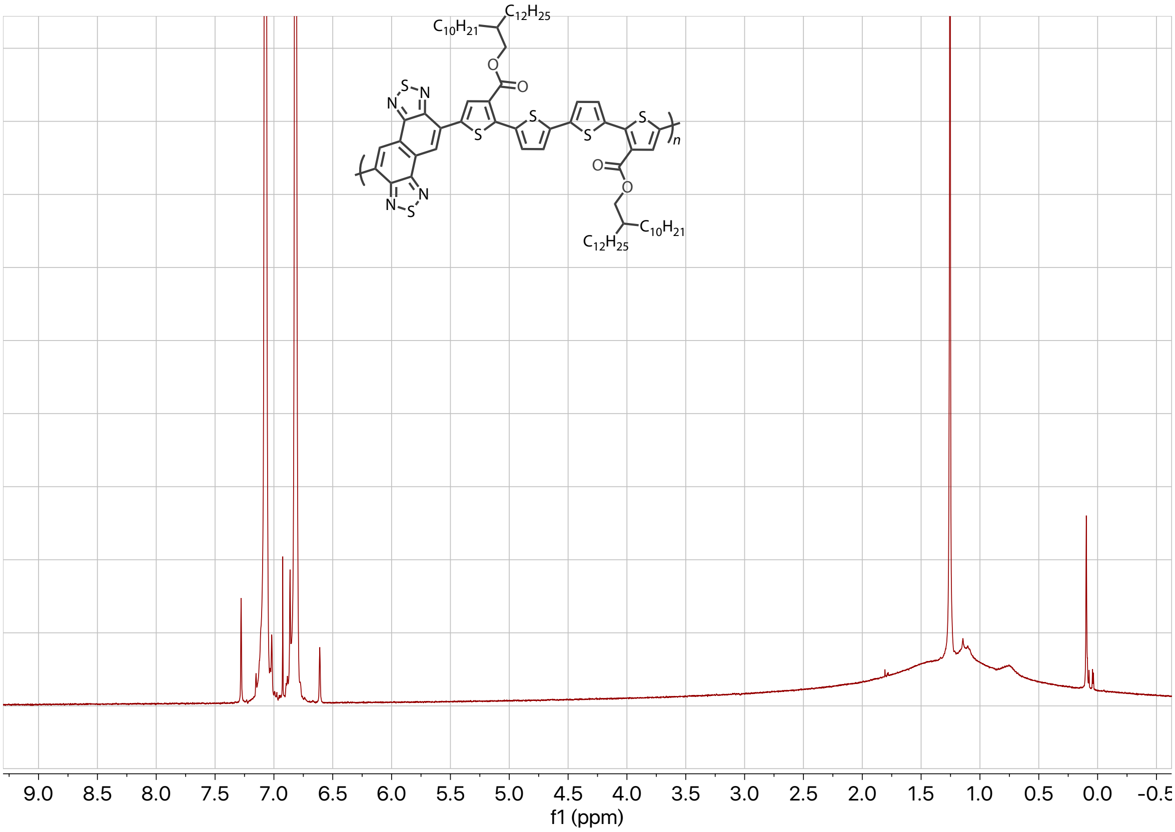

^{1}H-NMR spectra of **oF2** (upper) and **E-F0** (lower)

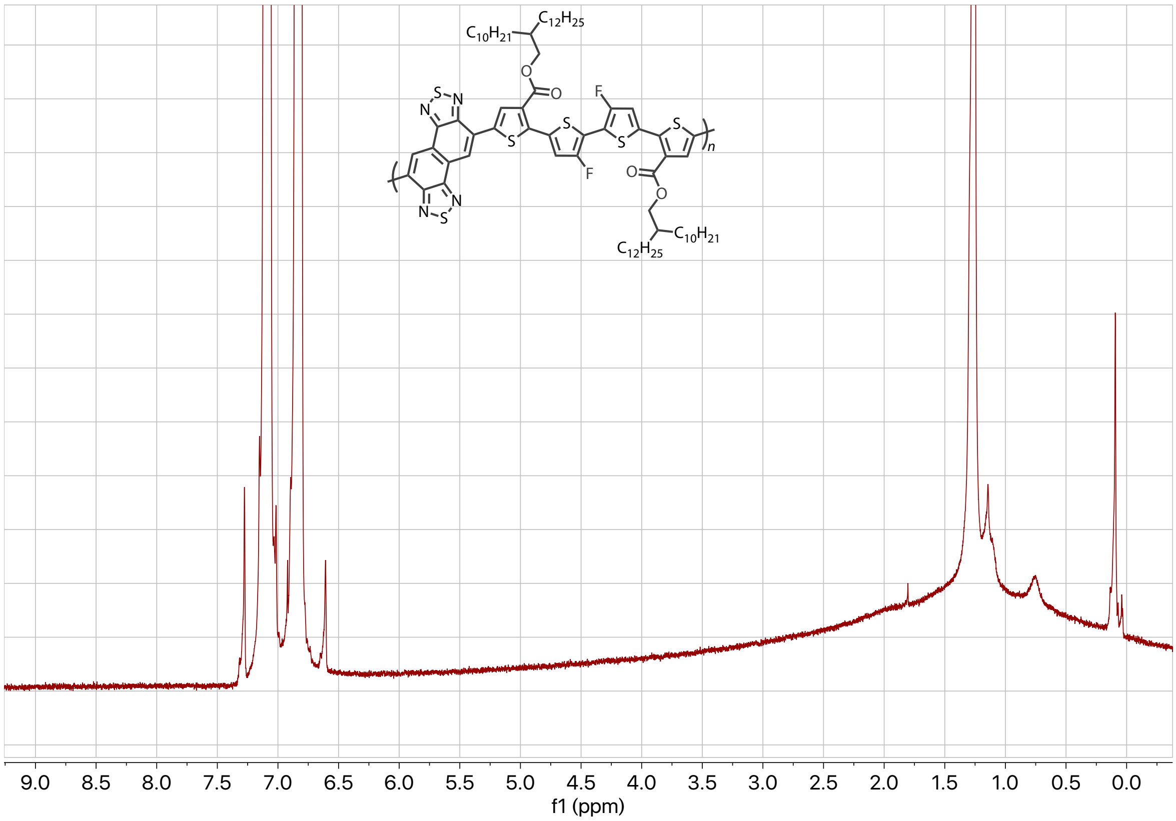

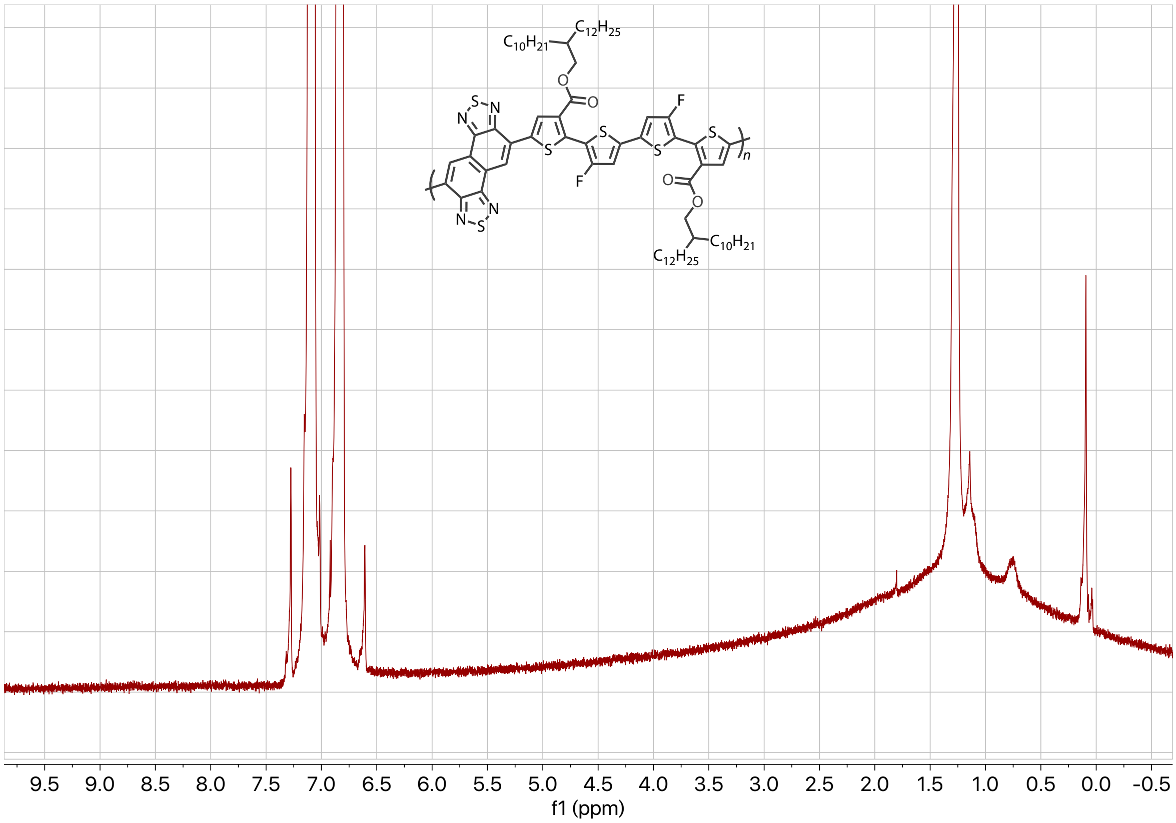

^{1}H-NMR spectra of **E-iF2** (upper) and **E-oF2** (lower)

3. Instrumentation

Differential scanning calorimetry (DSC) analysis was carried out with an EXSTAR DSC7020 (SII Nanotechnology, Inc.) at a cooling and heating rate of 10 °C min^{-1}. Cyclic voltammetry (CV) was carried out with an ALS Electrochemical Analyzer Model 612D in acetonitrile containing tetrabutylammonium hexafluorophosphate (Bu_4NPF_6, 0.1 M) as the supporting electrolyte at a scan rate of 100 mV s^{-1}. The counter and working electrodes were made of Pt, and the reference electrode was Ag/Ag$^+$. All potentials were calibrated with the standard ferrocene/ferrocenium redox couple (Fc/Fc$^+$: $E^{1/2}$ = +0.30 V measured under the identical conditions). E_{HOMO} and E_{LUMO} were calculated with the following equations:

E_{HOMO} (eV) = –4.50 – E_{ox}

E_{LUMO} (eV) = –4.50 + E_{red}

where E_{ox} and E_{red} are the onset oxidation and reduction potential of cyclic voltammograms, respectively. Photoelectron yield spectroscopy (PYS) in air was performed by a photoelectron spectrometer, model AC-2 (Riken Keiki Co., Ltd). Experimental setup for the low-energy inverse photoelectron spectroscopy (LEIPS) is described elsewhere.[S6-S8] The sample specimen was introduced into the vacuum chamber evacuated to 2 × 10^{-7} Pa and incident to an electron beam. In order to avoid the sample damage, the kinetic energy of incident electrons was restricted to less than 4 eV and the electron current densities ranged between 10^{-6} and 10^{-5} A cm^{-2}. Under these experimental conditions, no discernible change in the LEIPS spectra were observed due to the sample damage. The emitted photons were analyzed by a photon detector consisting of an optical bandpass filter and a photomultiplier tube. The center wavelength of the bandpass filter was 260, 285, and 335 nm. The vacuum level was determined as the onset energy of the sample current. For both PYS and LEIPS, the thin film samples were prepared by spin-coating from the chlorobenzene solution of the polymers (1–2 g L^{-1}) on the ITO-coated glass substrate. UV–vis absorption spectra were measured using a Shimadzu UV-3600 spectrometer. Photoluminescence spectra were measured with a fluorescence spectrometer

(Horiba Jobin Yvon, NanoLog) equipped with a photomultiplier tube (Hamamatsu, R928P) and a liquid-nitrogen-cooled InGaAs near-infrared array detector (Horiba Jobin Yvon, Symphony II) under ambient atmosphere. The excitation wavelength was set at 500 and 800 nm to estimate the quenching efficiency of donor and acceptor, respectively. Transmission electron microscopy (TEM) was conducted on JEM-2021 (JEOL).

Grazing incidence wide-angle X-ray diffraction (GIXD) measurements

GIXD measurements were performed with a HUBER multi-axis diffractometer installed in the beamline BL46XU at SPring-8. The X-ray beam from the undulator was monochromatized by a double-crystal Si(111) monochromator. The X-ray energy was 12.39 keV (λ = 1 Å), and the X-ray beam size was 40 μm (height) × 300 μm (width) at the sample position. The diffraction from the samples was detected by a two-dimensional (2D) X-ray photon counting pixel detector (PILATUS 300 K). The X-ray beam incidence angle was set to 0.12 °, and the camera length (sample-to-detector distance) was set to 174 mm. The neat films were prepared by spin-coating the material solution in chloroform on the ZnO coated ITO glass, where the substrate size was 1 cm × 1 cm. For the blend films, the photoactive layer on the OPV cells were directly used for the measurements. The measurements were performed in air at room temperature. The exposure time was 1 s, and no irradiation damage was observed on the samples. The coherence length (L_C) was estimated from the simplified Scherrer's equation, $L = 2\pi/\mathrm{fwhm}$,[S9] where fwhm is the full-width at half maximum of the lamellar and π–π stacking diffraction peaks. All the profiles were fitted to *pseudo*-Voigt functions, so that the overlapped peaks were separated, and linear backgrounds were subtracted. Note that fwhm was not corrected for the resolution function typically caused by the sample size.

Photovoltaic Cell Fabrication and Measurement.

ITO substrates were first pre-cleaned sequentially by sonicating in a detergent bath with de-

ionized water, acetone, and isopropanol at room temperature, and in a boiled isopropanol bath each for 10 min, and then baked at 120 °C for 10 min in air. The substrates were then subjected to a UV/ozone treatment at room temperature for 20 min. A ZnO layer was prepared by spin-coating (at 1200 rpm) from a dispersion of ZnO nanoparticles in chloroform. The photoactive layers were deposited in a glove box (KOREA KIYON, KK-011AS-EXTRA) by spin-coating a polymer/acceptor solution. The details of the spin-coating condition for each polymer are shown below.

oF2/PC$_{61}$BM: The *o*-dichlorobenzene solution containing 10 g L^{-1} of **oF2** with PC$_{61}$BM (oF2:PC$_{61}$BM = 1:2 w/w) was kept at 90 °C for 30 min, and the hot solution was directly spin-coated on the substrate, which was pre-heated at 90 °C, at 600 rpm for 20 s.

E-F0/PC$_{61}$BM and E-oF2: The *o*-dichlorobenzene solution containing 6 g L^{-1} of the polymer with PC$_{61}$BM (polymer:PC$_{61}$BM = 1:2 w/w) was kept at 100 °C for 30 min, and the hot solution was directly spin-coated on the substrate, which was pre-heated at 100 °C, at 600 rpm for 20 s.

E-iF2/PC$_{61}$BM: The *o*-dichlorobenzene solution containing 3 g L^{-1} of **E-iF2** with PC$_{61}$BM (**E-iF2**:PC$_{61}$BM = 1:2 w/w) was kept at 160 °C for 30 min, and the hot solution was directly spin-coated on the substrate, which was pre-heated at 160 °C, at 600 rpm for 20 s.

F0/Y12, oF2/Y12, E-F0/Y12, and E-oF2/Y12: The chlorobenzene solution containing 5 g L^{-1} of the polymer with Y12 (polymer:Y12 = 1:1.2 w/w) was kept at 100 °C for 1 h, and the hot solution was directly spin-coated on the substrate, which was pre-heated at 100 °C, at 1200 rpm for 20 s. The thin film was then thermally annealed at 90 °C for 5 min.

iF2/Y12: The chlorobenzene solution containing 4 g L^{-1} of **iF2** with Y12 (**iF2**:Y12 = 1:1.2 w/w) was kept at 100 °C for 1 h, and the hot solution was directly spin-coated on the substrate, which was pre-heated at 100 °C, at 1200 rpm for 20 s. The film was then thermally annealed at 90 °C for 5 min.

E-iF2/Y12: The chlorobenzene solution containing 2 g L^{-1} of **E-iF2** with Y12 (**E-iF2**:Y12 = 1:1.2 w/w) was kept at 120 °C for 1 h, and the hot solution was directly spin-coated on the

substrate, which was pre-heated at 120 °C, at 1200 rpm for 20 s. The film was then thermally annealed at 90 °C for 5 min.

The films were transferred into a vacuum evaporator (ALS Technology, E-100J) connected to the glove box. MoO_x (7.5 nm) and Ag (100 nm) were deposited sequentially through a shadow mask by thermal evaporation under approximately 10^{-5} Pa, where the active area of the cells was 0.1256 cm^2. *J–V* characteristics of the cells were measured with a Keithley 2400 source measure unit in nitrogen atmosphere under 1 Sun (AM1.5G) conditions using a solar simulator (SAN-EI Electric, XES-40S1). The light intensity for the *J–V* measurements was calibrated with a reference PV cell (Konica Minolta AK-100 certified by the National Institute of Advanced Industrial Science and Technology, Japan). EQE spectra were measured with a Spectral Response Measuring System (Soma Optics, Ltd., S-9241). The thickness of the active layer was measured with an ET4000 (Kosaka Laboratory, Ltd.). More than 10 different substrates (four active areas each) were made and measured to collect the photovoltaic properties.

Hole-Only Devices Fabrication and Measurement

The pre-cleaned ITO glass were coated with PEDOT:PSS by spin-coating at 5000 rpm for 30 s. The active layer was then deposited by spin-coating as described in the "Photovoltaic fabrication and measurement" section. The substrates were transferred into the vacuum evaporator, and the MoO_x (7.5 nm) and Ag (150 nm) layers were deposited sequentially through a shadow mask. The *J–V* characteristics were measured in the range of 0–7 V using a Keithley 2400 source measure unit in the dark in the glovebox. The hole mobility (μ) was calculated by fitting the *J–V* curves on the basis a space charge limited current (SCLC) model described by the following Mott–Gurney equation:

$$J = (8/9)\,\varepsilon_r\varepsilon_0\mu(V^2/L^3)$$

where ε_r is the relative dielectric constant of the polymer, ε_0 is the permittivity of free space, V

$= V_{appl} - V_{bi}$, and L is the active layer thickness. Here, V_{appl} is the applied voltage to the device and V_{bi} is the built-in voltage due to the difference in work function of the two electrodes (determined to be 0.1 V for hole-only device and 0 V for electron-only device, respectively). The dielectric constant ε_r is assumed to be 3, which is a typical value for semiconducting polymers.

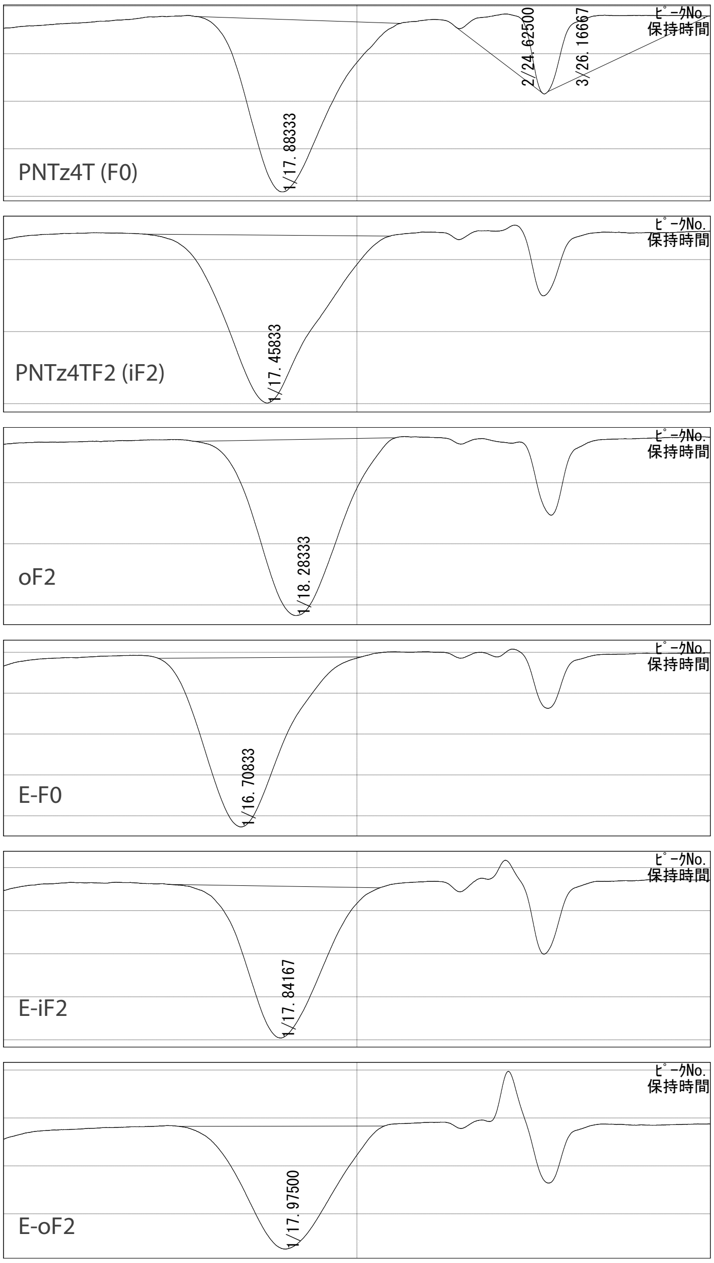

Figure S1. GPC charts for the polymers measured in *o*-dichlorobenzene at 140 °C.

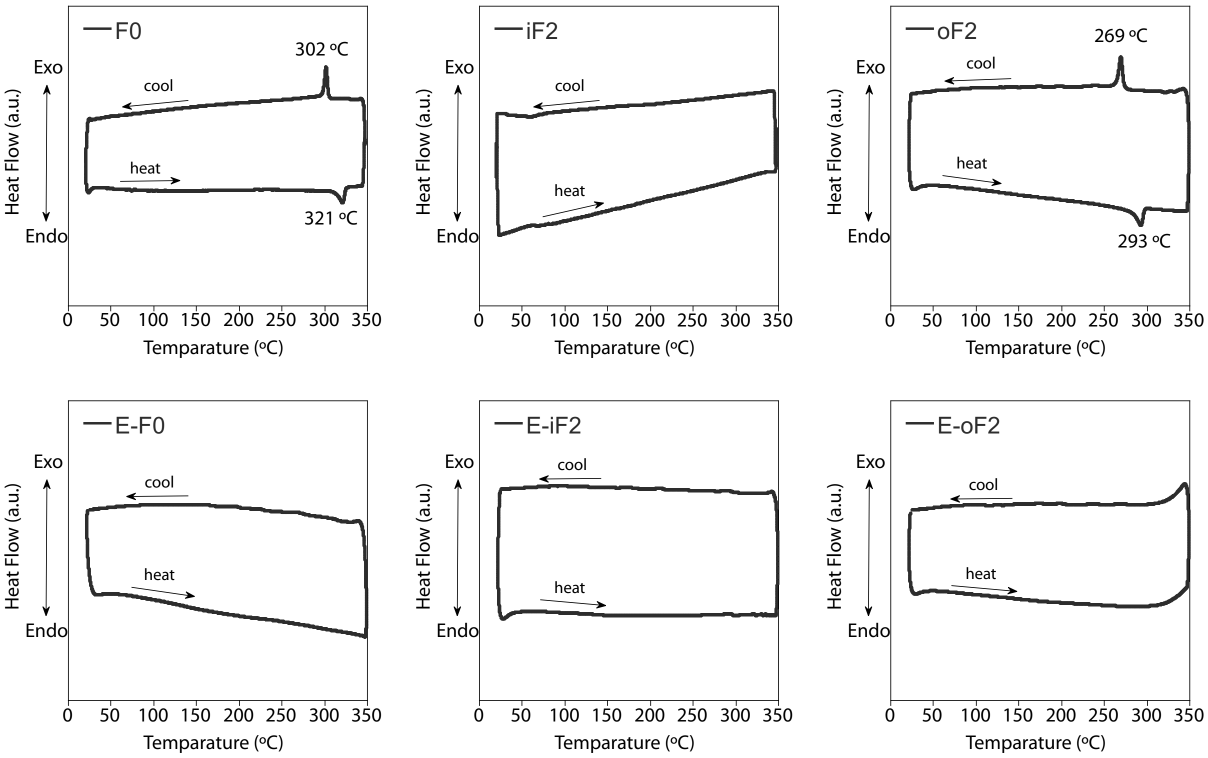

Figure S2. DSC curves of the polymers.

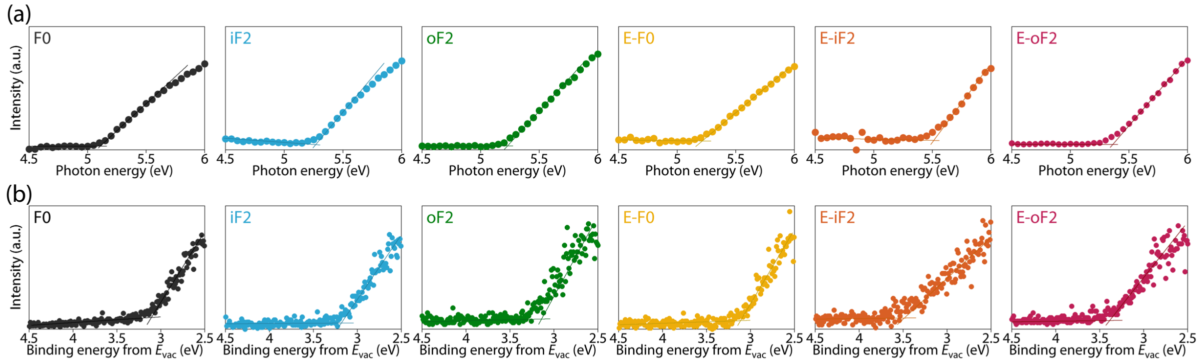

Figure S3. (a) Photoelectron yield spectra and (b) low-energy inverse photoelectron spectra of the polymers in the thin film.

Table S1. HOMO and LUMO energy levels determined by photoelectron yield spectroscopy (PYS) and low-energy inverse photoelectron spectroscopy (LEIPS), respectively. The transport gaps and exciton binding energies are calculated from the HOMO and LUMO energy levels.

Polymer	E_{HOMO} [eV][a)]	E_{LUMO} [eV][b)]	Transport gap [eV][c)]	Exciton binding energy [eV][d)]
**F0**	–5.09	–3.12	1.97	0.42
**iF2**	–5.27	–3.18	2.09	0.48
**oF2**	–5.20	–3.14	2.06	0.45
**E-F0**	–5.22	–3.11	2.11	0.55
**E-iF2**	–5.52	–3.51	2.01	0.34
**E-oF2**	–5.36	–3.39	1.97	0.36

[a)]HOMO energy level determined by PYS; [b)]LUMO energy levels determined by LEIPS; [c)]$E_{LUMO} – E_{HOMO}$; [d)] $(E_{LUMO} – E_{HOMO}) – E_g^{opt}$.

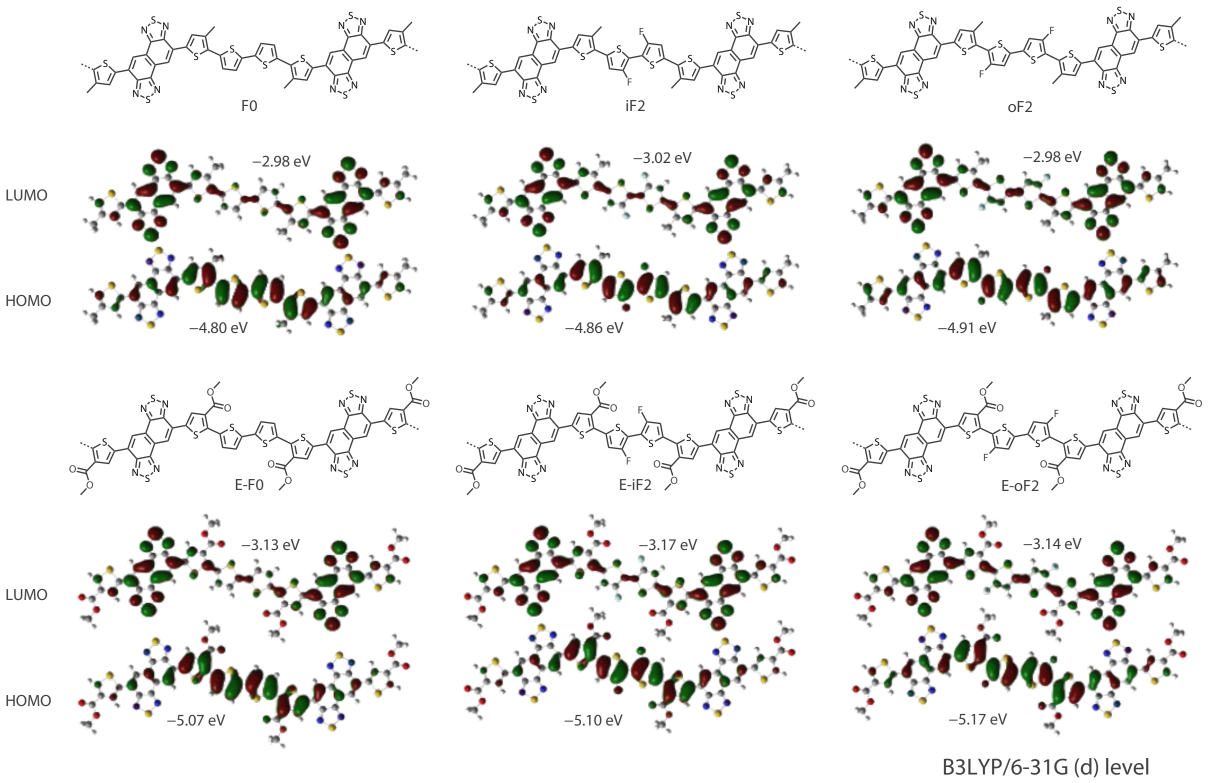

Figure S4. Optimized backbone geometry, HOMO and LUMO geometry, and HOMO and LUMO energy level for the model compounds calculated by the DFT method at the B3LYP/6-31G(d) level. All the alkyl groups were replaced with the methyl groups to simplify the calculation.

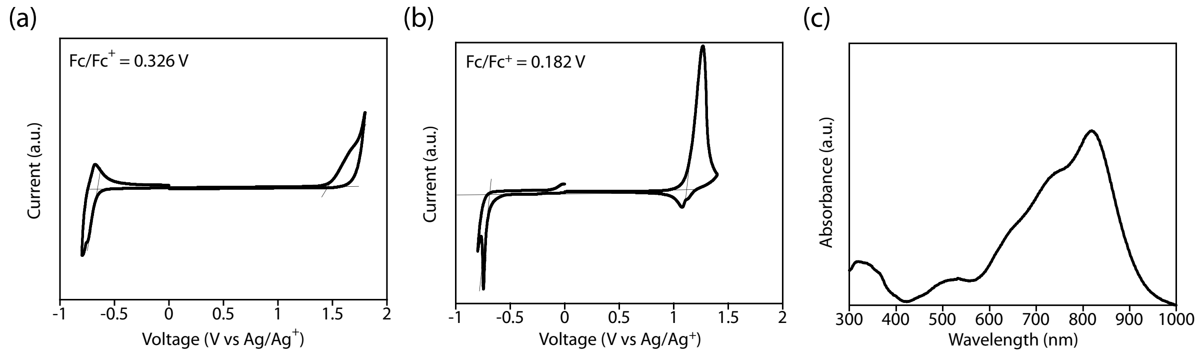

Figure S5. Cyclic voltammograms of (a) $PC_{61}BM$ and (b) Y12 in the thin film. (c) UV–vis absorption spectrum of Y12 in the thin film.

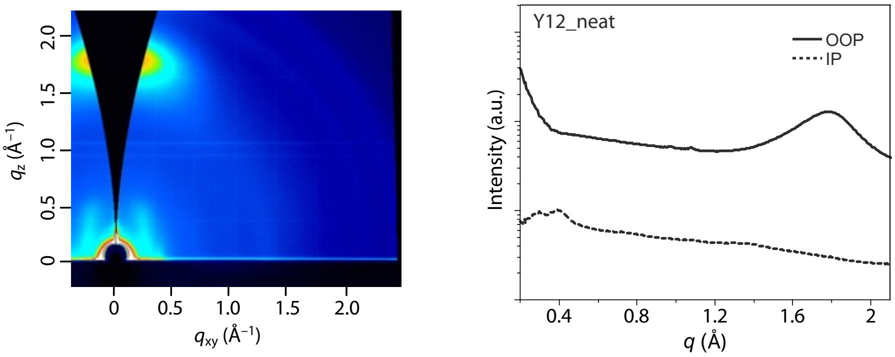

Figure S6. 2D GIXD patterns and cross-sectional diffraction profiles of Y12 neat film.

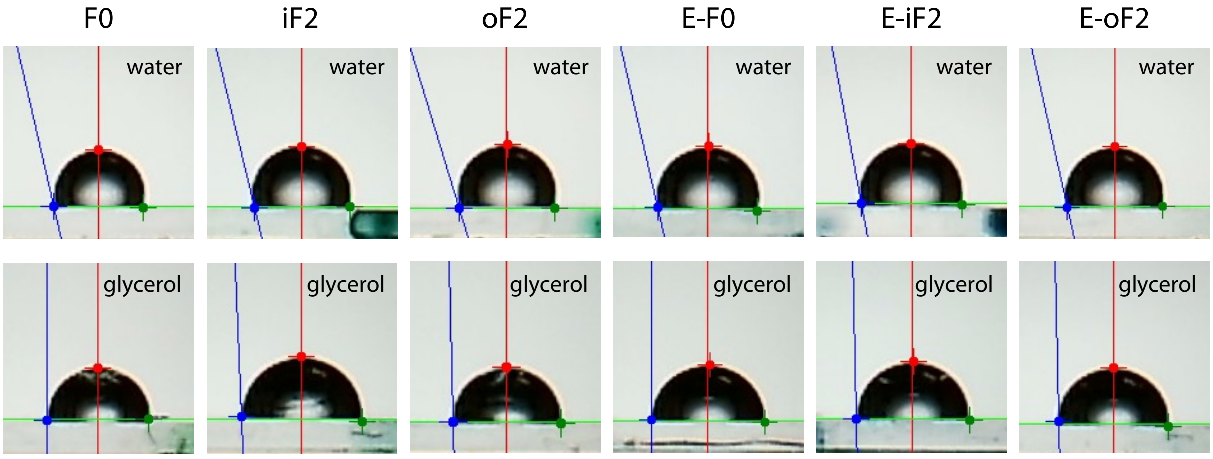

Figure S7. Contact angle measurements for the polymer films.

Table S2. Contact angle of water and glycerol for the neat films and the surface energy parameters

Material	θ_{water} [a)]	$\theta_{glycerol}$ [a)]	γ [mN m^{-1}] [b)]	$\sqrt{\gamma_D} - \sqrt{\gamma_A}$ [c)]	
				$PC_{61}BM$	Y6
**F0**	103.7	89.9	22.4	1.08	0.69
**iF2**	105.5	91.8	19.9	1.72	1.20
**oF2**	103.6	93.1	21.7	1.24	0.81
**E-F0**	103.7	90.5	21.8	1.20	0.78
**E-iF2**	104.2	90.4	20.9	1.42	0.95
**E-oF2**	102.0	90.9	22.2	1.13	0.71
$PC_{61}BM$	76.0	96.0	33.3	–	–
Y6	93.6	75.6	21.2	–	–

[a)]Contact angle of water and glycerol. [b)]γ: surface energy of the material. [c)]A parameter for the Flory–Huggins interfacial energy χ, defined as $\chi = k(\sqrt{\gamma_D} - \sqrt{\gamma_A})^2$, where γ_D and γ_A are surface energy of the donor (polymer) and the acceptor ($PC_{61}BM$ or Y6), respectively.

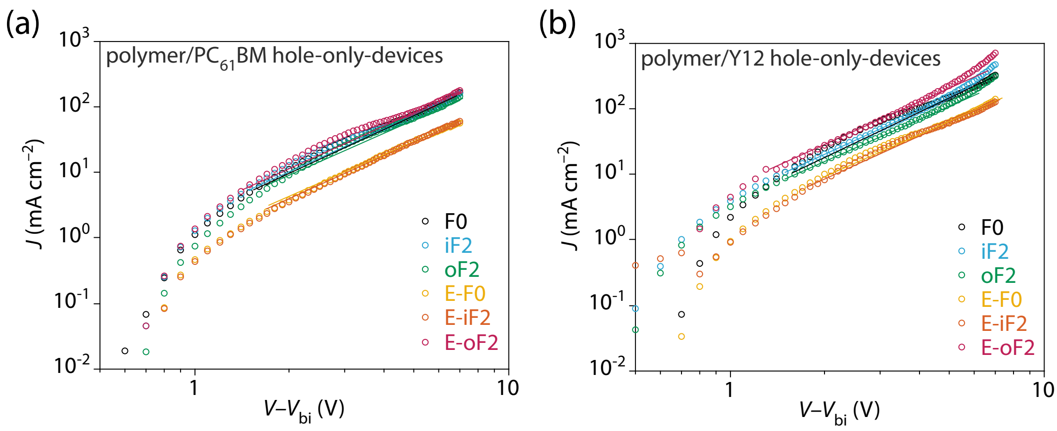

Figure S8. *J–V* curves of the hole-only devices for (a) polymer/$PC_{61}BM$ blends and (b) polymer/Y12 blends.

Table S3. Hole mobilities evaluated on the basis of the SCLC model.

Blend	μ [cm^2 V^{-1} s^{-1}]	Blend	μ [cm^2 V^{-1} s^{-1}]
**F0**/$PC_{61}BM$	1.1×10^{-3}	**F0**/Y12	8.5×10^{-4}
**iF2**/$PC_{61}BM$	9.5×10^{-4}	**iF2**/Y12	8.8×10^{-4}
**oF2**/$PC_{61}BM$	9.1×10^{-4}	**oF2**/Y12	8.0×10^{-4}
**E-F0**/$PC_{61}BM$	4.5×10^{-4}	**E-F0**/Y12	3.2×10^{-4}
**E-iF2**/$PC_{61}BM$	3.9×10^{-4}	**E-iF2**/Y12	2.9×10^{-4}
**E-oF2**/$PC_{61}BM$	1.2×10^{-3}	**E-oF2**/Y12	1.0×10^{-3}

References

[S1] I. Osaka, M. Shimawaki, H. Mori, I. Doi, E. Miyazaki, T. Koganezawa, K. Takimiya, *J. Am. Chem. Soc.* **2012**, 134, 3498.

[S2] K. Kawashima, T. Fukuhara, Y. Suda, Y. Suzuki, T. Koganezawa, H. Yoshida, H. Ohkita, I. Osaka, K. Takimiya, *J. Am. Chem. Soc.* **2016**, 138, 10265.

[S3] K. Kawashima, E. Miyazaki, M. Shimawaki, Y. Inoue, H. Mori, N. Takemura, I. Osaka, K. Takimiya, *Polym. Chem.* **2013**, 4, 5224.

[S4] D. E. Seitz, S.-H. Lee, R. N. Hanson, J. C. Bottaro, *Synth. Commun.* **1983**, 13, 121.

[S5] M. Kim, W.-T. Park, S. A. Park, C. W. Park, S. U. Ryu, D. H. Lee, Y.-Y. Noh, T. Park, *Adv. Funct. Mater.* **2019**, 29, 1805994.

[S6] H. Yoshida, *Chem. Phys. Lett.* **2012**, 539-540, 180.

[S7] H. Yoshida, *Rev. Sci. Instrum.* **2014**, 85, 016101.

[S8] H. Yoshida, *J. Electron Spectrosc. Relat. Phenom.* **2015**, 204, 116.

[S9] R.-J. Roe, *Methods of X-Ray and Neutron Scattering in Polymer Science*, Oxford University Press, Oxford, **2010**.
